# YY2/BUB3 Axis promotes SAC Hyperactivation and Inhibits Colorectal Cancer Progression via Regulating Chromosomal Instability

**DOI:** 10.1002/advs.202308690

**Published:** 2024-04-29

**Authors:** Rendy Hosea, Wei Duan, Ian Timothy Sembiring Meliala, Wenfang Li, Mankun Wei, Sharon Hillary, Hezhao Zhao, Makoto Miyagishi, Shourong Wu, Vivi Kasim

**Affiliations:** ^1^ Key Laboratory of Biorheological Science and Technology, Ministry of Education, College of Bioengineering Chongqing University Chongqing 400045 P. R. China; ^2^ The 111 Project Laboratory of Biomechanics and Tissue Repair, College of Bioengineering Chongqing University Chongqing 400044 P. R. China; ^3^ Department of Gastrointestinal Surgery, Chongqing University Cancer Hospital Chongqing University Chongqing 400030 P. R. China; ^4^ Life Science Innovation, School of Integrative and Global Majors University of Tsukuba Tsukuba Ibaraki 305‐0006 Japan; ^5^ Chongqing Key Laboratory of Translational Research for Cancer Metastasis and Individualized Treatment, Chongqing University Cancer Hospital Chongqing University Chongqing 400030 P. R. China

**Keywords:** chromosomal instability (CIN), drug resistance, mitosis, residual tumor cells, yin yang 2 (YY2)

## Abstract

Spindle assembly checkpoint (SAC) is a crucial safeguard mechanism of mitosis fidelity that ensures equal division of duplicated chromosomes to the two progeny cells. Impaired SAC can lead to chromosomal instability (CIN), a well‐recognized hallmark of cancer that facilitates tumor progression; paradoxically, high CIN levels are associated with better therapeutic response and prognosis. However, the mechanism by which CIN determines tumor cell survival and therapeutic response remains poorly understood. Here, using a cross‐omics approach, YY2 is identified as a mitotic regulator that promotes SAC activity by activating the transcription of budding uninhibited by benzimidazole 3 (*BUB3*), a component of SAC. While both conditions induce CIN, a defect in YY2/SAC activity enhances mitosis and tumor growth. Meanwhile, hyperactivation of SAC mediated by YY2/BUB3 triggers a delay in mitosis and suppresses growth. Furthermore, it is revealed that YY2/BUB3‐mediated excessive CIN causes higher cell death rates and drug sensitivity, whereas residual tumor cells that survived DNA damage‐based therapy have moderate CIN and increased drug resistance. These results provide insights into the role of SAC activity and CIN levels in influencing tumor cell survival and drug response, as well as suggest a novel anti‐tumor therapeutic strategy that combines SAC activity modulators and DNA‐damage agents.

## Graphical abstract

CIN is a hallmark of cancer and can drive tumorigenesis; paradoxically, high CIN correlates with better prognosis in clinical patients. The underlying cause of this paradox is still unclear. This study identifies the novel role of YY2 in modulating BUB3‐mediated SAC activity and CIN, as well as YY2/BUB3‐mediated SAC activity and CIN levels role in determining tumor cell fates.

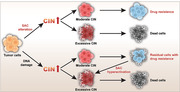

## 1 Introduction

Chromosomal instability (CIN) is characterized by an increased rate of chromosomal structural and numerical abnormalities due to aberrant segregation. Chromosomal abnormalities cause significant fitness costs to normal cells, leading to various disorders, including metabolic alterations, proteotoxic stress, cell cycle arrest, and senescence.^[^ ^1^ ^]^ Furthermore, it is the most common cause of spontaneous abortion and severe developmental defects.^[^ ^2^ ^]^ Despite these deleterious effects, CIN is a well‐recognized cancer hallmark, with ≈90% of tumors displaying complex karyotypes, including structural and numerical CIN.^[^ ^3^ ^]^ Moreover, CIN can promote tumor initiation and is associated with metastasis, therapeutic resistance, and poor survival outcomes.^[^ ^4^, ^5^, ^6^, ^7^, ^8^ ^]^ However, paradoxically, high CIN levels are associated with improved prognosis and better therapeutic responses, such as those using DNA‐damage agents, thus demonstrating a tumor suppressive function.^[^ ^9^, ^10^, ^11^ ^]^

Impaired activity of the spindle assembly checkpoint (SAC), one of the major cell cycle checkpoints, is a major cause of CIN.^[^ ^12^ ^]^ As a checkpoint at the metaphase–anaphase transition point, it ensures that sister chromatids are segregated equally into the two progeny cells and is thus the last checkpoint to guarantee mitotic fidelity through the passage of correct, intact genetic information to the progeny cells.^[^ ^13^ ^]^ SAC activity prevents premature cohesion cleavage, sister chromatid segregation, and mitotic exit by suppressing securin and cyclin B ubiquitination/proteasomal degradation before all sister chromatids are lined up at the equator and attached to spindle microtubules.^[^ ^13^ ^]^ Defect in SAC triggers premature sister chromatid segregation and mitotic exit, leading to increased CIN and faster mitosis, thereby serving as a driving force for tumorigenesis.^[^ ^14^, ^15^ ^]^ Similarly, SAC hyperactivation also induces CIN; however, it leads to mitotic delay and tumor suppression.^[^ ^16^, ^17^ ^]^ The role of SAC hyperactivation in tumorigenesis remains poorly understood. Furthermore, the reasons underlying the different outcomes related to SAC defects and hyperactivation remain to be explored.

Although mutations in SAC genes are rare, alterations in SAC gene expression are frequently found in tumor cells, suggesting a crucial role for transcriptional regulation in aberrant SAC activity in tumor cells.^[^ ^18^, ^19^ ^]^ However, the mechanisms underlying SAC transcriptional regulation have not yet been fully elucidated. In this study, we utilized a cross‐omics approach to search for potential transcriptional regulators of SAC genes and identified yin yang 2 (YY2) as a novel *budding uninhibited by benzimidazole 3* (*BUB3*) transcriptional regulator. Our results showed that YY2/BUB3 axis positively modulates SAC activity, prolongs mitotic time, and suppresses colorectal cancer (CRC) cells survival. However, despite of their opposite effects on SAC activity and CRC cells survival, both YY2/BUB3 deficiency and overexpression lead to increased CIN. We further revealed that different CIN degrees induced by YY2/BUB3 alterations is crucial for determining CRC cell survival and drug response, notably, excessive CIN level leads to cell death while moderate CIN is beneficial for CRC cells. Finally, our results showed a strong correlation between moderate CIN and drug resistance, such as that found in residual tumor cells resistant to DNA damage‐inducing agents and/or SAC activation. In contrast, the induction of excessive CIN in those cells by *YY2* overexpression sensitizes them to DNA damage‐inducing agents, suggesting a novel anti‐tumor therapeutic approach by combining SAC activator and DNA damage‐inducing agents.

## 2 Results

### 2.1 Identification of Transcriptional Regulators Modulating SAC

**Figure 1:**
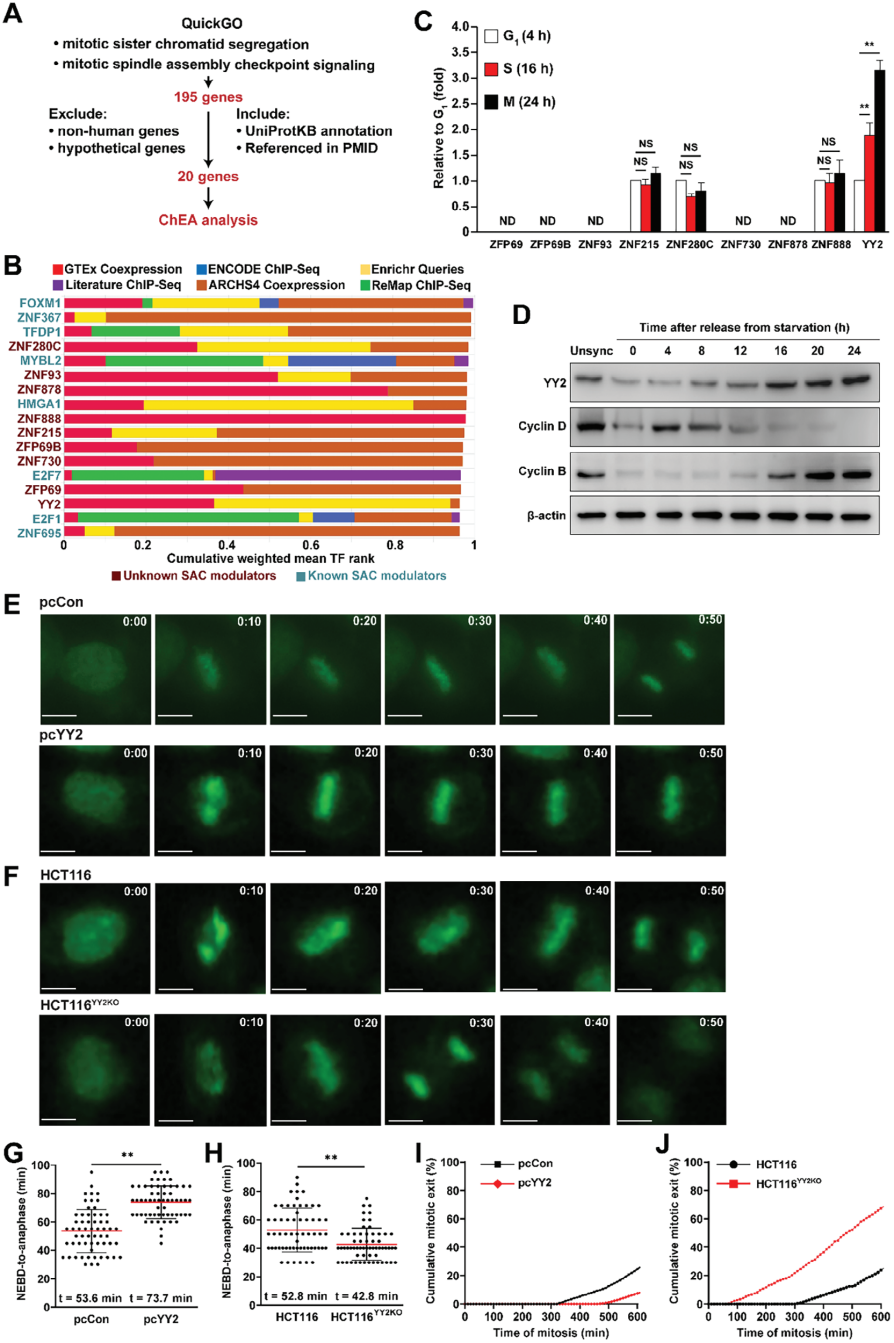
Identification of novel transcriptional regulators modulating SAC. A) Schematic diagram of screening strategy for potential novel transcriptional regulators modulating SAC. B) Top 17 potential TFs modulating SAC obtained from ChEA analysis. C) mRNA expression levels of potential novel SAC modulators at each cycle phase, as examined using qRT‐PCR. D) YY2, cyclin D, and cyclin B protein expression levels in HCT116 cells at indicated time‐points after serum starvation release, as determined by western blotting. E–F) Mitotic time of *YY2*‐overexpressed HCT116 cells (E) and HCT116^YY2KO^ cells (F), as determined using time‐lapse microscopy. Representative images (scale bars: 20 µm) are shown. G–H) Scatter plots showing the time‐length from nuclear envelope breakdown (NEBD) to anaphase of *YY2*‐overexpressed HCT116 cells (G) and HCT116^YY2KO^ cells (H) (total n = 60, pooled from three independent experiments). I,J) Time‐lapse analysis of duration of mitosis in *YY2*‐overexpressed HCT116 cells (I) and HCT116^YY2KO^ cells (J) arrested with nocodazole (final concentration: 100 ng mL^−1^; total n = 150, pooled from three independent experiments). Cells transfected with pcCon, wild‐type HCT116, or unsynchronized cells were used as controls. β‐actin was used for qRT‐PCR normalization and as western blotting loading control. Quantification data are shown as mean ± SD. All data were obtained from three independent experiments. *P* values were calculated by one‐way analysis of variance (ANOVA). pcCon: pcEF9‐Puro; Unsync: unsynchronized; **P < 0.01; NS: not significant; ND: not detected.

To identify a novel modulator of the SAC and subsequently tumor cell CIN, we screened genes involved in both “mitotic sister chromatid segregation” and “mitotic spindle assembly checkpoint signaling” from the Gene Ontology (GO) database (QuickGO, http://www.ebi.ac.uk/QuickGO; GO numbers: 0000070 and 0007094; respectively) and obtained 195 genes. After excluding non‐human‐origin and hypothetical genes, we further screened genes that have been reviewed and annotated using the manual‐curation process in UniProtKB and referenced based on a PubMed Unique Identifier (PMID). The 20 genes identified in this screening were subjected to transcription factor (TF) enrichment analysis using enrichment analysis version 3 (ChEA3, https://maayanlab.cloud/chea3/), a web server application developed to conduct TF enrichment analysis utilizing gene set libraries from published omics assay data extracted from multiple sources^[^ ^20^ ^]^ (**Figure** **1A**). Among the top 17 TFs obtained, eight TFs were known SAC modulators, thus confirming the validity of this screening method;^[^ ^21^, ^22^, ^23^, ^24^ ^]^ whereas nine TFs were potential novel SAC modulators (Figure 1B).

TFs that regulate the cell cycle usually exhibit oscillating levels during cell cycle progression.^[^ ^25^ ^]^ Thus, we first determined the timing of each cell cycle phase by synchronizing HCT116 CRC cells in the G_0_/G_1_ phase using serum starvation (Figure S1A, Supporting Information). The percentage of cells in the G_0_/G_1_ phase decreased from 78.12% immediately after serum starvation release to 14.11% 16 h later, whereas cells in the S phase increased from 10.20% to a peak at 76.89% after 16 h, a timing necessary for HCT116 cells to progress into S phase after starvation‐induced G0 phase by serum starvation as reported by previous study.^[^ ^26^ ^]^ Meanwhile, cells in the G_2_/M phase reached 55.69% at 24 h after serum starvation release. Cell cycle‐dependent expression analysis of the nine novel SAC modulators revealed that significant expression of ZFP69, ZFP69B, ZNF93, ZNF730, and ZNF878 could not be detected in any cell cycle state, while those of ZNF215, ZNF280C, and ZNF888 did not show significant differences during cell cycle progression. Meanwhile, YY2 mRNA expression clearly increased during cell cycle progression and peaked during the M phase (Figure 1C). A more detailed time‐course investigation showed that YY2 mRNA expression peaked at 20 h (Figure S1B, Supporting Information), whereas its protein level started to increase significantly at 16 h and peaked at 24 h after serum starvation release (Figure 1D). Meanwhile, the levels of G_1_ and G_2_/M phase cyclins, specifically cyclins D and B, peaked at 4 h and 24 h after serum starvation release, respectively, further confirming the relationship between YY2 expression and the M phase.

SAC delays mitotic exit by stabilizing securin and suppressing APC/C activity. To further explore the role of YY2 in regulating SAC activity and subsequently mitotic exit, we altered *YY2* expression in HCT116 cells, a near‐diploid human CRC cell line with a mitotic time similar to that of normal cells and considered CIN‐negative cells (Figure S1C,D, Supporting Information).^[^ ^27^ ^]^ As shown by time‐lapse microscopic images, *YY2* overexpression delayed the mitosis, whereas *YY2* knockout significantly accelerated it (Figure 1E,F, Videos S1–S4, Supporting Information). Measurement of mitotic time revealed that *YY2* overexpression prolonged the mitotic time from 53.6 min to 73.7 min, while *YY2* knockout significantly shortened it from 52.8 min to 42.8 min (Figure 1G,H). Notably, when *YY2* was reintroduced into *YY2*‐knocked out cells to the level similar to control, the mitotic time was also restored to wild‐type level, further confirming the function of YY2 in regulating mitosis (Figure S1E–G, Supporting Information). Next, we modulated *YY2* expression and synchronized cells in the M phase using nocodazole, a metaphase‐arresting SAC activator. *YY2* overexpression significantly slowed down the degradation of securin and cyclin B, an event that marks mitotic exit (Figure S2A,B, Supporting Information), whereas knocking out *YY2* accelerated their degradation (Figure S2C,D, Supporting Information). Moreover, to further characterize the role of YY2 in regulating SAC activity, we analyzed the mitotic time of nocodazole‐treated cells. Compared to nocodazole‐treated control cells, *YY2*‐overexpressed cells exhibited prolonged mitotic time under nocodazole treatment (Figure S2E, Supporting Information), while *YY2‐*knocked out cells underwent shorter mitotic arrest (Figure S2F, Supporting Information). Furthermore, to confirm that the observed prolonged mitosis in *YY2*‐overexpressed cells was due to increase in SAC strength rather than mere activation of SAC, we investigated the ability of *YY2*‐overexpressed cells treated with nocodazole to sustain an extended mitotic arrest.^[^ ^28^ ^]^ *YY2* overexpression led to stronger SAC activity, as demonstrated by fewer cells that exit mitosis under constant SAC activation (Figure 1I); meanwhile, *YY2* knockout exerted the opposite (Figure 1J). These results solidified the role of YY2 in promoting SAC activity. Together, our results revealed that YY2, of which the expression oscillates during the cell cycle and reaches its peak in the M phase, is a novel SAC modulator that controls mitotic progression.

### 2.2 *YY2‐*Mediated SAC Regulation is Crucial for its Tumor Suppressive Effect

**Figure 2:**
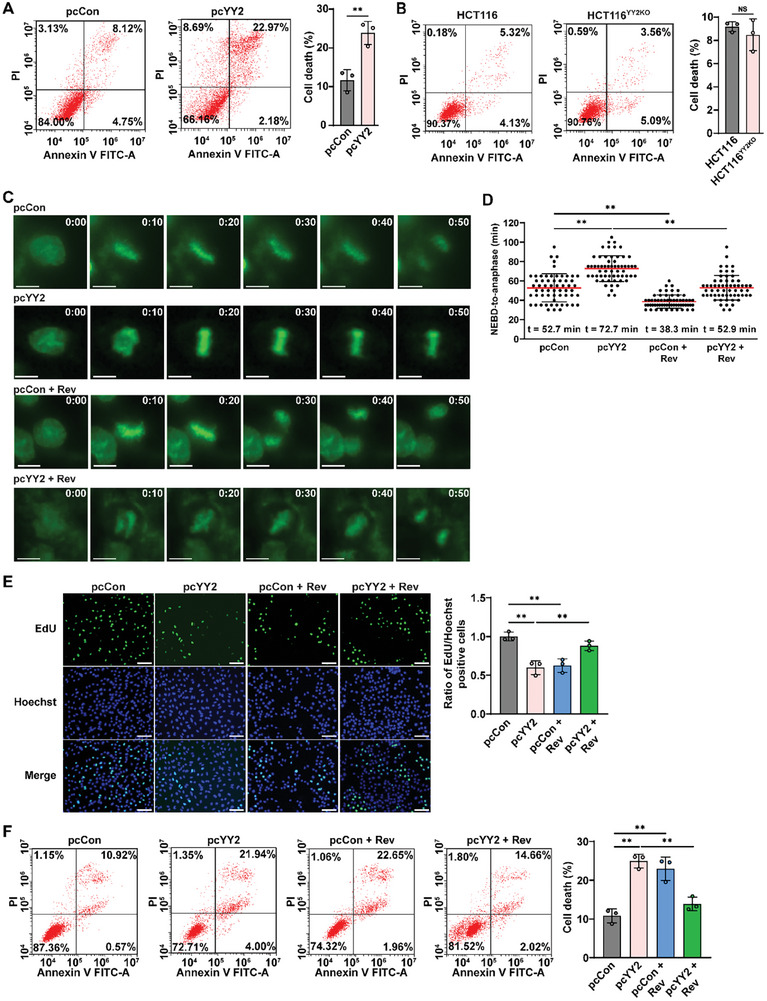
YY2 suppresses tumorigenesis by activating SAC activity. A,B) Cell death rate of *YY2*‐overexpressed HCT116 (A) and HCT116^YY2KO^ cells (B), as examined using Annexin V/PI staining and flow cytometry. C,D) Mitotic time of *YY2*‐overexpressed, reversine‐treated HCT116 cells, as determined using time‐lapse microscopy. Representative images (C; scale bars: 20 µm) and scatter plot showing the time‐length from NEBD to anaphase (D; total n = 60, pooled from three independent experiments) are shown. E) Proliferation potential of *YY2*‐overexpressed, reversine‐treated HCT116 cells, as determined using EdU‐incorporation assay. Representative images (left; scale bars: 200 µm) and ratio of proliferative cells (right; each dot represents the mean value of three technical replicates) are shown. F) Cell death rate of *YY2*‐overexpressed, reversine‐treated HCT116 cells, as examined using Annexin V/PI staining. Cells transfected with pcCon or wild‐type HCT116 cells were used as controls. Quantification data are shown as mean ± SD. All data were obtained from three independent experiments. *P* values were calculated by one‐way ANOVA. pcCon: pcEF9‐Puro; Rev: reversine (final concentration: 0.2 µM); **P < 0.01; NS: not significant.

YY2 is a zinc‐finger protein and has been reported to have a tumor suppressive function.^[^ ^29^, ^30^, ^31^, ^32^ ^]^ A comparative analysis using clinical CRC tissues and corresponding normal adjacent tissues revealed a significant decrease in YY2 mRNA and protein levels in CRC tissues (Figure S3A,B, Supporting Information). Furthermore, *YY2* overexpression significantly suppressed the viability and colony‐formation potential of HCT116 cells (Figure S3C,D, Supporting Information). As a decrease in cell viability might be due to a decrease in proliferation, increase in cell death, or both, we next examined the effect of YY2 on HCT116 cell proliferation and cell death. *YY2* overexpression robustly suppressed CRC cell proliferation, as indicated by the decrease in EdU‐positive cells (Figure S3E, Supporting Information), while increasing the cell death rate (**Figure** **2**A), suggesting that YY2 may exert its tumor suppressive function most plausibly by decreasing cell proliferation and promoting cell death. In contrast, the viability, colony‐formation potential, and proliferation potential of HCT116^YY2KO^ cells were significantly higher than those of wild‐type cells (Figure S3F–H, Supporting Information). Interestingly, *YY2* knockout did not significantly affect the cell death rate (Figure 2B), most plausibly because of the overall low cell death rate. Notably, the cell death rate increased significantly when *YY2* was reintroduced into *YY2*‐knocked out cells in excessive level, further confirming that *YY2* overexpression could induce cell death (Figure S3I, Supporting Information). It is noteworthy that proper level SAC activity is important to prevent event that can induce cell death, such as chromosome missegregation.

To examine whether YY2 exerts its tumor suppressive effect by regulating SAC activity, we treated *YY2*‐overexpressed HCT116 cells with reversine, a SAC activity inhibitor.^[^ ^33^, ^34^ ^]^ In control cells, reversine treatment reduced cyclin B and securin accumulation to levels significantly lower than those in control cells; meanwhile, in *YY2*‐overexpressed cells, it reduced cyclin B and securin accumulation induced by *YY2* overexpression to levels near to those in control cells (Figure S3J, Supporting Information). Furthermore, reversine treatment re‐suppressed mitotic time prolonged by *YY2* overexpression from 72.7 min to 52.9 min, which was also similar to control (Figure 2C,D, Video S5, Supporting Information). These results indicated that reversine treatment restored YY2‐induced SAC hyperactivation to a level similar to control. Accordingly, while reversine treatment suppressed the proliferation, viability, and colony‐formation potential of control HCT116 cells, it restored these potentials in *YY2*‐overexpressed HCT116 cells (Figure 2E; Figure S3K,L, Supporting Information). Similarly, reversine treatment increased cell death rate in control HCT116 cells, while suppressing that of *YY2* overexpressed cells (Figure 2F). These results not only reveal that proper level of SAC activity is crucial to support cell survival but also YY2‐mediated SAC activation and mitotic regulation are crucial for exerting its tumor suppressive effect.

### 2.3 YY2 Regulates SAC by Directly Activating BUB3 Transcription

**Figure 3:**
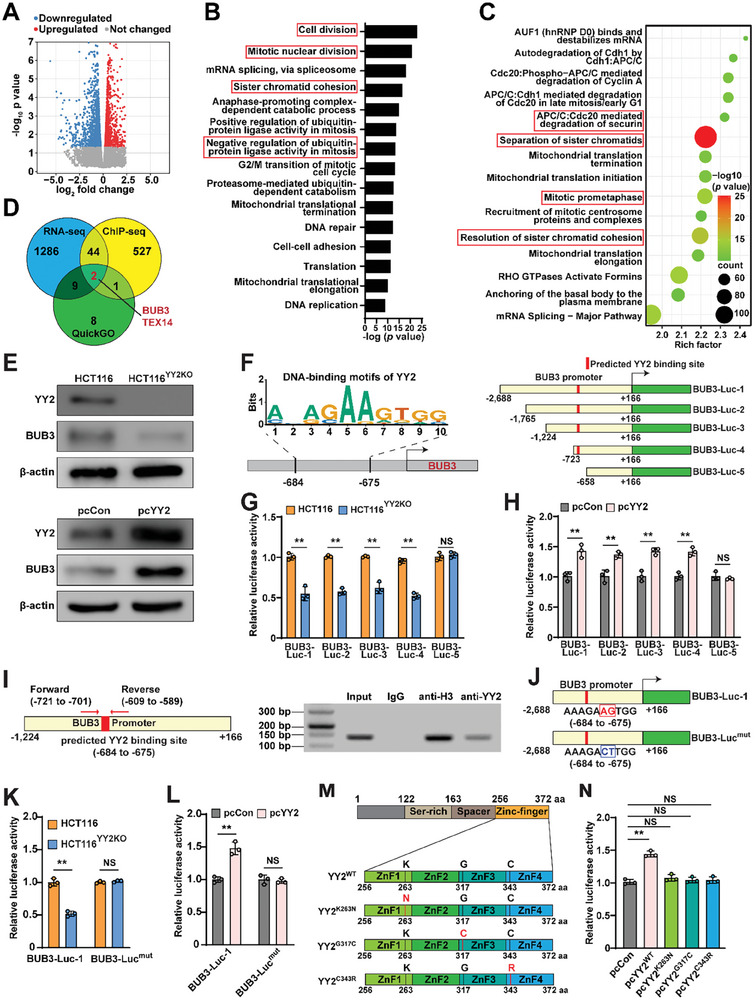
YY2 directly regulates *BUB3* transcription. A) Volcano plot of log2 fold‐change versus adjusted *P* value for gene expression changes (fold‐change > 1.01‐fold; *P* value < 0.05) in *YY2*‐overexpressed HCT116 cells, as analyzed by RNA‐seq. B–C) GO B) and Reactome C) enrichments of differentially expressed genes in *YY2*‐overxpressed HCT116 cells. D) Overlapping genes upregulated by YY2 based on RNA‐seq, predicted YY2 target genes identified by ChIP‐seq, and QuickGO screening. E) BUB3 protein level in *YY2*‐overexpressed HCT116 and HCT116^YY2KO^ cells, as determined using western blotting. F–H) Schematic diagram (F) as well as relative luciferase activities of *BUB3* reporter vectors in HCT116^YY2KO^ cells (G) and *YY2*‐overexpressed HCT116 cells (H). I) Binding capacity of YY2 to the predicted region in the *BUB3* promoter, as determined using ChIP assay. Location of the primer pair used for PCR are shown. J–L) Schematic diagram (J) as well as relative luciferase activities of BUB3‐Luc^mut^ in HCT116^YY2KO^ cells (K) and *YY2*‐overexpressed HCT116 cells (L). M,N) Schematic diagram of *YY2* mutants overexpressing vectors (M) and relative luciferase activity of BUB3‐Luc‐1 in HCT116 cells overexpressing indicated *YY2* mutants (N). Cells transfected with pcCon or wild‐type HCT116 cells were used as controls. β‐actin was used as western blotting loading control. Quantification data are shown as mean ± SD. All data were obtained from three independent experiments. *P* values were calculated by one‐way ANOVA. pcCon: pcEF9‐Puro; **P < 0.01; NS: not significant.

To analyze the molecular mechanism underlying YY2‐mediated regulation of SAC activity, we performed a cross‐omics analysis to identify its potential transcriptional target. To this end, RNA‐sequencing (RNA‐seq) data obtained in our previous study (https://www.ncbi.nlm.nih.gov/geo/query/acc.cgi?acc=GSE184138) using *YY2*‐overexpressed HCT116 cells^[^ ^29^ ^]^ were analyzed for differentially expressed genes (DEGs; **Figure** **3**A). The DEGs (fold‐change > 1.01; *P* < 0.05) were then analyzed for enriched GO terms and Reactome pathways using the Database for Annotation, Visualization, and Integrated Discovery (DAVID, https://david.ncifcrf.gov/). GO analysis indicated that *YY2* overexpression enriched genes involved in cell division, mitotic nuclear division, sister chromatid cohesion, and negative regulation of ubiquitin‐protein ligase activity in mitosis (Figure 3B); meanwhile, Reactome analysis revealed the enrichment of APC/C‐Cdc20‐mediated degradation of securin, separation of sister chromatids, mitotic prometaphase, and resolution of sister chromatid cohesion (Figure 3C), indicating a possible prominent role of YY2 in regulating sister chromatid segregation.

To identify potential YY2 transcriptional targets that regulate SAC activity, we overlapped DEGs identified by RNA‐seq with those identified by chromatin immunoprecipitation (ChIP)‐sequencing using an anti‐YY2 antibody reported previously,^[^ ^30^ ^]^ as well as genes obtained from QuickGO screening (Figure 1A). Two genes, *BUB3* and *TEX14*, were identified as potential YY2 direct transcriptional targets (Figure 3D). BUB3 is a component of the SAC complex that regulates sister chromatid segregation and mitotic progression;^[^ ^13^ ^]^ meanwhile, TEX14 is required for the formation of intercellular bridges during meiosis.^[^ ^35^ ^]^ In accordance with previous studies,^[^ ^35^, ^36^ ^]^ analysis of the TEX14 expression profiles using GTEx v8 expression data across 29 tissues revealed its testis specificity (Figure S4A, Supporting Information). Furthermore, absolute qRT‐PCR results showed that the copy number of *TEX14* was very low in HCT116 cells (Figure S4B, Supporting Information). Meanwhile, BUB3 mRNA increased nearly two‐fold upon *YY2* overexpression (Figure S4C, Supporting Information), whereas its protein level was positively correlated with YY2 in *YY2*‐knock‐down and *YY2*‐overexpressed HCT116 cells (Figure 3E). Moreover, to avoid the effect of YY2 oscillation during cell cycle, we arrested the HCT116 cells at G_0_/G_1_ phase using serum starvation and investigated the effect of altering YY2 expression on BUB3. The results consistently demonstrated that *YY2* alteration positively correlates with BUB3 expression level, further confirming the direct regulation of YY2 on BUB3 expression (Figure S4D,E, Supporting Information).

Next, we analyzed the *BUB3* promoter using JASPAR (http://jaspar.genereg.net/),^[^ ^37^ ^]^ and identified a potential YY2‐binding site at −684 to −675 of its promoter region (Figure 3F). To confirm the role of this binding site in YY2‐mediated regulation of *BUB3* transcriptional activity, we constructed reporter vectors comprising the −2688 to +166 region (BUB3‐Luc‐1), −1765 to +166 region (BUB3‐Luc‐2), −1224 to +166 region (BUB3‐Luc‐3), −723 to +166 region (BUB3‐Luc‐4), and −658 to +166 region (BUB3‐Luc‐5) of the *BUB3* promoter (Figure 3F). *YY2* knockout robustly suppressed the transcriptional activities of BUB3‐Luc‐1, BUB3‐Luc‐2, BUB3‐Luc‐3, and BUB3‐Luc4, which contained the predicted YY2‐binding site, but not that of BUB3‐Luc‐5, which lacked the predicted binding site (Figure 3G). Concomitantly, *YY2* overexpression promoted the activities of BUB3‐Luc‐1, BUB3‐Luc‐2, BUB3‐Luc‐3, and BUB3‐Luc4, but not that of BUB3‐Luc‐5 (Figure 3H). These results suggest that the −723 to −659 region of the *BUB3* promoter is crucial for YY2‐mediated regulation of its transcriptional activity. Furthermore, the result of a ChIP assay using an anti‐YY2 antibody revealed that YY2 could bind to the −721 to −589 region of the *BUB3* promoter (Figure 3I). Subsequently, mutations at the predicted YY2‐binding site on the *BUB3* promoter (AAAGA*AG*TGG to AAAGA*CT*TGG; BUB3‐Luc^mut^, Figure 3J) abolished the YY2‐mediated regulation of its transcriptional activity (Figure 3K,L). To further confirm YY2 transcriptional regulation on *BUB3*, we used mutant *YY2* overexpression vectors with a zinc finger mutation constructed previously based on cancer genomics data set from the cBioportal database (Figure 3M).^[^ ^29^, ^38^ ^]^ Our results showed that these mutant YY2 failed to regulate *BUB3* promoter transcriptional activity (Figure 3N). Together, these results showed that YY2 is a transcriptional regulator of *BUB3* that binds to the −684 to −675 site of its promoter region.

To elucidate the role of BUB3 in YY2‐induced SAC activity and prolonged mitosis, we first analyzed the effect of *BUB3* overexpression on mitotic time. The level of BUB3 in cells transfected with *BUB3* overexpression vectors was confirmed (Figure S4F,G, Supporting Information). Similar to that of *YY2* overexpression, *BUB3* overexpression in HCT116 cells prolonged the mitotic time from 51.3 min to 80.5 min (Figure S4H,I and Video S6, Supporting Information) and slowed the degradation rates of cyclin B and securin proteins (Figure S4J–L, Supporting Information). *BUB3* overexpression restored the length of HCT116 cells mitotic time, which was shortened to 43.3 min by *YY2* knockout, to a level similar to that observed in the control (50 min and 52.9 min, respectively; Figure S5A–C and Video S7, Supporting Information). Next, we constructed two shRNA expression vectors targeting different sites of BUB3 and selected shBUB3‐2 (refers as shBUB3 hereafter), which had a higher suppressive effect, for further experiments (Figure S5D,E, Supporting Information). Similar to the trend observed after knocking out *YY2*, knocking down *BUB3* shortened the mitotic time of HCT116 cells from 52.7 min to 40.5 min and resuppressed the mitotic time, which was prolonged by *YY2* overexpression, from 71.4 min to 46.8 min (Figure S5F–H and Videos S8,S9, Supporting Information). Accordingly, *BUB3* knockdown restored HCT116 cell viability and cell death, which was suppressed by *YY2* overexpression (Figure S5I,J, Supporting Information). These results confirmed that YY2 regulates SAC and subsequently, mitotic progression and tumorigenic potential by enhancing *BUB3* expression.

### 2.4 Both *YY2* Knockout and Overexpression induce CIN

**Figure 4:**
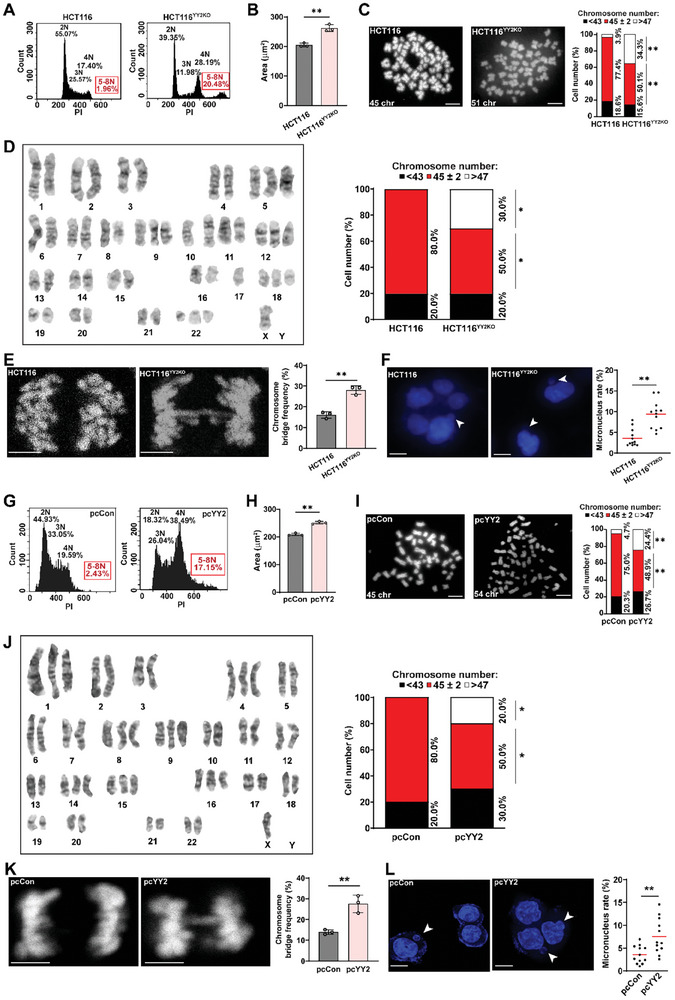
Both *YY2* knockout and overexpression induces CIN. A) DNA content in HCT116^YY2KO^ cells, as analyzed using PI staining and flow cytometry. B) Average nuclei size of DAPI‐stained HCT116^YY2KO^ cells (each dot represents average nuclei size from one independent experiment, with total 100 cells/group). C) Chromosome number per cell in HCT116^YY2KO^ cells, as analyzed using metaphase spread. Representative images (scale bars: 10 µm) and percentage of cells with indicated chromosome number (total cells counted: 50 cells/group, pooled from three independent experiments) are shown. D) Single karyotype analysis of HCT116^YY2KO^ cells. Representative images and percentage of cells with indicated chromosome number (total cells counted: 10 cells/group, pooled from three independent experiments) are shown. E) Chromosome bridge frequency in HCT116^YY2KO^ cells. Representative images (scale bars: 5 µm) and quantification results (each dot represents chromosome bridge frequency from one independent experiment, with total 100 mitotic‐cells/group) are shown. F) Micronucleus rate in HCT116^YY2KO^ cells. Representative images of micronuclei (indicated by arrowheads; scale bars: 20 µm) and micronucleus rate (ratio of micronuclei number to total cell number; each dot represents micronucleus rate/slide with > 100 cells/slides; four technical replicates from three independent experiments) are shown. G–L) DNA content analysis (G), average nuclei size (H), chromosome number per cell (I), single karyotype analysis (J), chromosome bridge frequency (K), and micronucleus rate (L) in *YY2*‐overexpressed HCT116 cells. Cells transfected with pcCon or wild‐type HCT116 cells were used as controls. Quantification data are shown as mean ± SD. All data were obtained from three independent experiments. *P* values were calculated by one‐way ANOVA. pcCon: pcEF9‐Puro; *P < 0.05; **P < 0.01.

Defects in SAC activity disrupt the mechanism that guarantees the proper arrangement of chromosomes at the equator and attachment to the mitotic spindle, making them prone to missegregation, and therefore, is a well‐known causal factor of CIN induction. Thus, we examined the effect of *YY2* silencing on CIN indicators. We observed significant increases in the frequency of cells with DNA content > 4N (**Figure** **4**A) as well as in nuclear size (Figure 4B) in HCT116^YY2KO^ cells, indicating the increase of the frequency of polyploid cells. Metaphase spread analysis results showed a significant increase in the karyotypic heterogeneity of HCT116^YY2KO^ cells, as nearly 80% of HCT116 cells had 45–47 chromosomes and fewer than 4% had more than 47 chromosomes, whereas only approximately 50% of HCT116^YY2KO^ cells had 45–47 chromosomes and the percentage of cells with more than 47 chromosomes increased to nearly 35% (Figure 4C). Single karyotype analysis further confirmed the increase of karyotypic heterogeneity in HCT116^YY2KO^, indicated by clones with gain or loss of chromosome (Figure 4D; Figure S6A, Supporting Information).

Failed sister chromatid segregation leads to the formation of partly unsegregated sister chromatids or chromosome bridges, of which DNA fragments are subsequently wrapped by a nuclear membrane‐like structure during cytokinesis to form a micronucleus, a small, nucleus‐like structure in the cytoplasm. *YY2* knockout significantly increased the chromosome bridge frequency (Figure 4E) and concomitantly increased the micronucleus rate (Figure 4F), which could lead to the formation of aneuploid cell. Meanwhile, *BUB3* overexpression could partially abrogate the frequencies of polyploid cells and chromosome bridges, as well as the micronucleus rate in HCT116^YY2KO^ cells, while reintroduction of *YY2* into *YY2*‐knocked out cells fully restored them (Figure S6B–D, Supporting Information). Together, these results suggest that defects in the YY2/BUB3 pathway could contribute to CIN induction in tumor cells, most plausibly due to impaired SAC activity.

Surprisingly, *YY2* overexpression, which induced SAC hyperactivation and mitotic delay, also increased the number of polyploid cells, nuclear size, karyotype heterogeneity, chromosome bridge frequency, and micronucleus rates (Figure 4G–L; Figure S6E, Supporting Information). These results suggest that *YY2* overexpression might also increase CIN.

Given that BUB3 is a component of SAC, we overexpressed *BUB3* and examined its effect on CIN. *BUB3* overexpression induced cell death (Figure S7A, Supporting Information) and increased CIN phenotypes (Figure S7B–E, Supporting Information). These results conform with previous studies showing that SAC activation could also induce CIN and furthermore, cell death.^[^ ^16^, ^39^ ^]^ To further confirm the causal relation between *YY2* overexpression‐induced SAC hyperactivation and CIN, we suppressed SAC activity in HCT116 cells overexpressing *YY2*. Suppression of *BUB3* or treatment with reversine significantly decreased the number of polyploid cells (Figure S8A,B, Supporting Information), chromosome bridge frequency (Figure S8C,D, Supporting Information), and micronucleus rate (Figure S8E,F, Supporting Information) in HCT116 cells overexpressing *YY2*, which is likely due to the suppression of SAC activity to near wild‐type level in these cells as shown in Figure S5F,G (Supporting Information) Together, our results clearly show that both *YY2* knockout and overexpression induced CIN.

### 2.5 CIN Levels Determine Tumor Cell Survival

**Figure 5:**
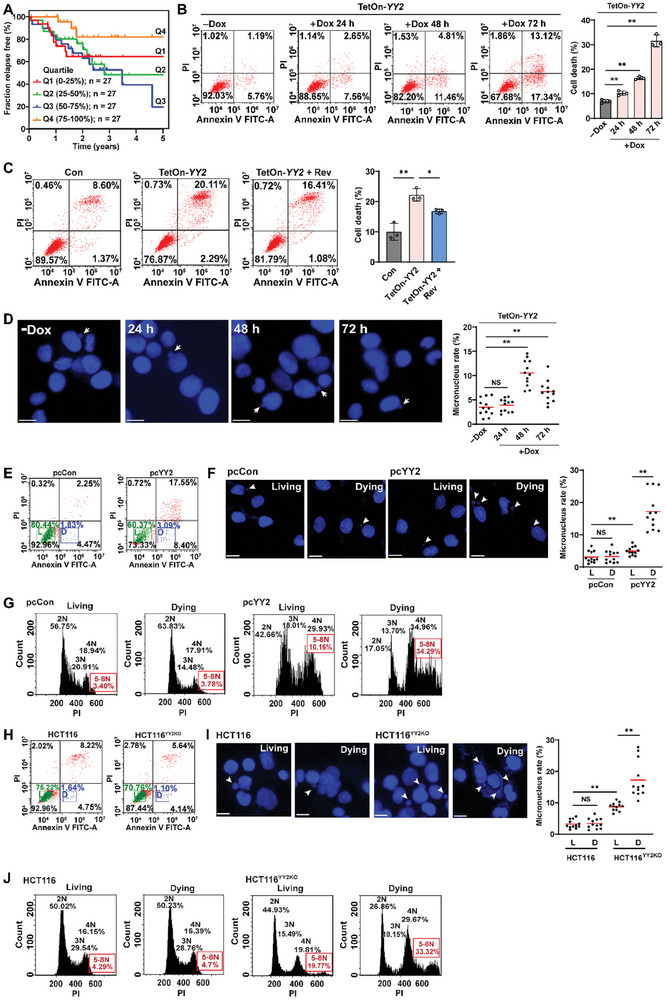
CIN level determines tumor cell survival. A) Kaplan‐Meier relapse‐free survival curves of 108 oxaliplatin‐treated CRC patients stratified by CIN70 score quartile. B,C) Cell death rate of HCT116 cells after *YY2* induction by doxycycline (B) and after reversine treatment (final concentration: 0.2 µM) (C), as examined using Annexin V/PI staining. D) Micronucleus rate in HCT116 cells after *YY2* induction by doxycycline. Representative images of micronuclei (indicated by arrowheads; scale bars: 20 µm) and micronucleus rate (ratio of micronuclei number to total cell number; each dot represents micronucleus rate/slide with > 100 cells/slides; four technical replicates from three independent experiments) are shown. E–G) Sorting (E), micronucleus rate (F), and DNA content (G) of living (L) and dying (D) *YY2*‐overexpressed HCT116 cells. H–J) Sorting (H), micronucleus rate (I), and DNA content (J) of living L) and dying (D) HCT116^YY2KO^ cells. Cells transfected with pcCon, wild‐type HCT116 cells, or HCT116 cells infected with lentivirus generated using pTRIPZ‐control (Con) were used as controls. Quantification data are shown as mean ± SD. All data were obtained from three independent experiments. *P* values were calculated by one‐way ANOVA. Scale bars: 20 µm; pcCon: pcEF9‐Puro; Dox: doxycycline (final concentration: 2 µg mL^−1^); **P < 0.01; NS: not significant.

The aforementioned results showing that both *YY2* knockout and overexpression induced CIN were intriguing, as *YY2* knockout suppressed SAC activity, shortened the mitotic time, and promoted HCT116 cell proliferation, whereas YY2 overexpression induced SAC hyperactivation, prolonged the mitotic time, enhanced cell death, and suppressed HCT116 cell proliferation. Meanwhile, clinical studies have shown that patients with high CIN have a better prognosis.^[^ ^9^, ^10^, ^11^ ^]^ Using TCGA CRC data set, we next performed correlation analysis between relapse‐free survival rate of CRC patients treated with oxaliplatin, a drug currently used as first‐line therapy for treating CRC in clinical practice,^[^ ^40^ ^]^ and the pretreatment levels of CIN70, a 70‐gene signature that has been established as surrogate of CIN.^[^ ^41^ ^]^ The results showed that the moderate CIN levels, i.e., the second and third quartiles, were associated with poor prognosis; meanwhile, high levels of CIN, i.e., the fourth quartile, were associated with better survival, suggesting that excessive CIN might be deleterious for tumors (**Figure** **5**A).

We next examined the survival of *YY2*‐overexpressed cells. To control the timing of *YY2* overexpression, we established a Tet‐On *YY2* overexpression system (Figure S9A,B, Supporting Information). The cell death rate increased in a time‐dependent manner after the induction of *YY2* overexpression (Figure 5B); while treatment with reversine cancelled it (Figure 5C), suggesting the correlation between *YY2* overexpression‐induced SAC hyperactivation and cell death. Given that *YY2* overexpression also led to increased CIN (Figure 4G–L), these results indicated the possibility that YY2‐induced cell death might be correlated with SAC hyperactivation‐induced CIN. However, unlike the cell death rate, the micronucleus rate started to decline after reaching its peak at 48 h post‐doxycycline treatment, leading to questions regarding the survival of cells with high CIN (Figure 5D). To examine the relationship between the CIN level and cell death, we stained the cells with Annexin V and propidium iodide (PI), and sorted the “dying cells” (Annexin V^+^/PI^−^), in which nuclear and chromosomal DNA fragmentation had not occurred and the chromosome could still be observed, as well as “living cells” (Annexin V^−^/PI^−^) (Figure 5E). Whereas there was no significant difference between the micronucleus rate of living and dying control cells, the micronucleus rate, as well as the number of cells with more than one micronucleus in dying *YY2*‐overexpressed HCT116 cells, was significantly higher than that in living *YY2*‐overexpressed cells (Figure 5F). Similarly, whereas there was no significant difference in the percentage of polyploid cells between living and dying control cells (3.40% and 3.78%, respectively), the percentage of polyploid cells in dying *YY2*‐overexpressed HCT116 cells was significantly higher than that in the corresponding living cells (34.29% and 10.16%, respectively; Figure 5G).

Furthermore, while there were only few dying cells in non‐treated HCT116^YY2KO^ cells, we confirmed that the micronucleus rate and percentage of polyploidy cells in those cells were significantly higher than in living HCT116^YY2KO^ cells (Figure 5H–J). Moreover, while knocking out *YY2* alone did not induce cell death and even benefits tumor cell proliferation, treatment with reversine, which could further weaken SAC activity as indicated by the shortened mitotic time (Figure S9C,D and Videos S10, Supporting Information), increased CIN as well as cell death in HCT116^YY2KO^ cells (Figure S9E,F, Supporting Information). Hence, these results further confirmed the association between cell death with high CIN. It is noteworthy that while excessive CIN, whether due to SAC hyperactivation or due to its hypoactivity, eventually leads to cell death, intrinsic characteristics of the cells, such as different gene expression profiles, might also contribute to the capacity of the cells to tolerate CIN stress, and eventually to their different outcomes such as in cell proliferation.^[^ ^42^, ^43^ ^]^ Indeed, we observed different expression profiles of genes related with DNA damage response and negative regulation of apoptosis in *YY2*‐overexpressed and *YY2*‐knocked out HCT116 cells (Figure S9G, Supporting Information). Nevertheless, while further investigations are needed to systematically analyze these differences, our results clearly suggest that cells with excessive CIN induced by either YY2‐mediated SAC hyperactivation or *YY2* knockout are more prone to death.

**Figure 6:**
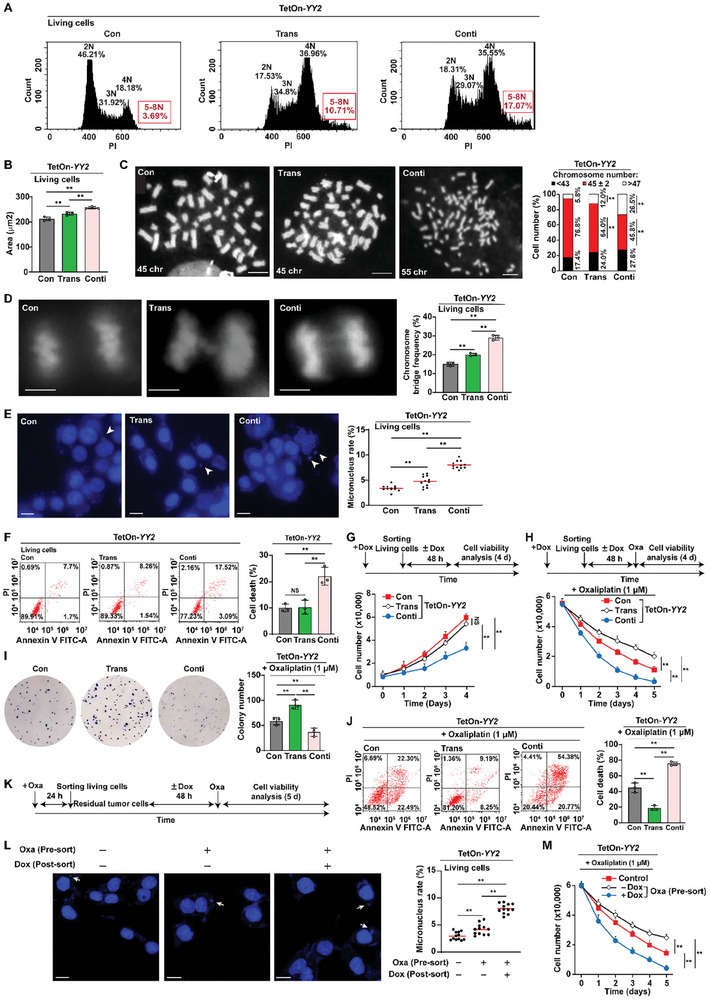
Residual tumor cells have moderate CIN and increased drug resistance. A) DNA content in HCT116 cells transiently overexpressing *YY2* (TetOn‐*YY2* Trans cells) 48 h after sorting, as analyzed using PI staining and flow cytometry. B) Average nuclei size of TetOn‐*YY2* Trans cells 48 h after sorting (each dot represents average nuclei size from one independent experiment, with total 100 cells/group). C) Chromosome number per cell in TetOn‐*YY2* Trans cells 48 h after sorting, as analyzed using metaphase spread. Representative images (scale bars: 10 µm) and percentage of cells with indicated chromosome number (total cells counted: 50 cells group^−1^, pooled from three independent experiments) are shown. D) Chromosome bridge frequency in TetOn‐*YY2* Trans cells 48 h after sorting. Representative images (scale bars: 5 µm) and quantification results (each dot represents chromosome bridge frequency from one independent experiment, with total 100 mitotic‐cells/group) are shown. E) Micronucleus rate of TetOn‐*YY2* Trans cells 48 h after sorting. Representative images of micronuclei (indicated by arrowheads; scale bars: 20 µm) and micronucleus rate (ratio of micronuclei number to total cell number; each dot represents micronucleus rate/slide with > 100 cells/slides; four technical replicates from three independent experiments) are shown. F,G) Cell death rate at indicated time‐points (F) and cell viability at 4 days after sorting (G) of TetOn‐*YY2* Trans cells. H–J) Viability (H), colony‐formation potential (I; each dot represents the mean value of three technical replicates), and cell death rate (J) of TetOn‐*YY2* Trans cells at indicated times, 10 days, and 5 days after oxaliplatin treatment, respectively. K–M) Schematic diagram (K), micronucleus rate (L), and viability after oxaliplatin treatment (M) of residual HCT116 cells infected with TetOn‐*YY2* lentivirus. Cells infected with control lentivirus generated from pTRIPZ‐control (Con) were used as controls. TetOn‐*YY2* Trans and Conti: cells infected with TetOn‐*YY2* lentivirus and treated with doxycycline only before sorting or continuously, respectively. Pre‐sort and post‐sort: pre‐sorting and post‐sorting. Quantification data are shown as mean ± SD. All data were obtained from three independent experiments. *P* values were calculated by one‐way ANOVA. Dox: doxycycline (final concentration: 2 µg mL^−1^); **P < 0.01; NS: not significant.

We further noticed that while the level of CIN in the living *YY2*‐overexpressed HCT116 cells was significantly lower than in the dying *YY2*‐overexpressed HCT116 cells, it was slightly higher than in living control cells, as indicated by the slightly increased micronucleus rate and percentage of polyploid cells (Figure 5F,G, respectively). Furthermore, as described above, moderate CIN level is correlated with poor prognosis (Figure 5A), thus raising the question regarding the characteristics of these moderate CIN cells. To examine their characteristics, we reversed *YY2* overexpression by withdrawing doxycycline after sorting the living cells to avoid continuous SAC hyperactivation (TetOn‐*YY2* Trans; Figure S10A,B, Supporting Information). Time‐dependent analysis showed that slight increase in micronucleus rate was maintained even at 96 h after doxycycline withdrawal (Figure S10C, Supporting Information), confirming that CIN is heritable and can be passed to the progeny cells as reported previously.^[^ ^44^ ^]^ Analysis of other CIN indicators 2 days after doxycycline withdrawal further confirmed this result, as the slightly increased CIN level was maintained in living TetOn‐*YY2* Trans cells (**Figure** **6**A–E). However, unlike the cells continuously overexpressing *YY2* (TetOn‐*YY2* Conti), whose viability and colony‐formation potential decreased and cell death rate increased, the viability, colony‐formation potential, and cell death rate of TetOn‐*YY2* Trans cells recovered to the levels similar to those of control despite that they have a certain level of CIN (Figure 6F,G; Figure S10D, Supporting Information). Meanwhile, while continuous *YY2* overexpression suppressed the viability and colony‐formation potential while increasing cell death rate (TetOn‐*YY2* Conti), these characteristics recovered to the levels similar to those of control in TetOn‐*YY2* Trans cells (Figure 6F,G; Figure S10D, Supporting Information). However, under DNA‐damage stress induced by oxaliplatin, TetOn‐*YY2* Trans cells demonstrated robustly higher viability and colony‐formation potential (Figure 6H,I). Moreover, the cell death rate of TetOn‐*YY2* Trans cells was significantly lower than HCT116 cells, which were CIN‐negative (Figure 6J), suggesting that CIN level before oxaliplatin treatment contribute to the observed differences in viability between cells with continuous and transient *YY2* overexpression. Meanwhile, suppressing CIN in TetOn‐*YY2* Conti cells using reversine decreased cell death and improved cell viability (Figure S10E–G, Supporting Information), indicating that reducing excessive CIN level by inhibiting SAC in SAC‐hyperactivated cells can enhance tumor cell drug resistance. Together, these data indicated the correlation between moderate CIN and tumor cell drug resistance.

Residual cells, which survived the anti‐tumor drug treatment and undetectable by standard morphological examination during the remission phase between the cycles of chemotherapy, has attracted attention as one of the crucial factors of tumor drug resistance and tumor recurrence, and thus as a hurdle for anti‐tumor therapy.^[^ ^45^ ^]^ The fact that living cells with YY2‐induced SAC hyperactivation possessed moderate CIN suggests the possibility that moderate CIN is also involved in drug resistance in residual tumor cells that survived treatment with DNA damage‐inducing agents. To test this possibility, we obtained residual tumor cells from CRC cells infected with TetOn‐*YY2* lentivirus by sorting living cells that survived oxaliplatin treatment, treated them with or without doxycycline to induce *YY2* overexpression, and examined their viability under oxaliplatin treatment (Figure 6K). Micronucleus assay revealed that without induction of *YY2* overexpression, residual cells that survived oxaliplatin treatment had moderate CIN level (Figure 6L, middle row); furthermore, these cells demonstrated improved DNA‐damage stress‐resistance compared to that in control cells (Figure 6M). Meanwhile, further induction of CIN by activating *YY2* overexpression in these residual cells induced excessive CIN (Figure 6L, right row), leading to better drug sensitivity (Figure 6M).

Together, our results demonstrated that YY2/SAC hyperactivation induces CIN in tumor cells, leading to the mortality of cells with excessive CIN. Moreover, our results suggest that moderate CIN, as observed in residual tumor cells surviving SAC hyperactivation or DNA damage‐inducing agent treatment, is crucial for drug resistance in these cells; while increasing CIN in these cells could improve their drug sensitivity. Hence, our findings highlight the crucial role of CIN levels in determining tumor cell survival and drug resistance.

### 2.6 *YY2*‐Induced Excessive CIN Sensitizes Tumor Cells to DNA Damage‐Inducing Agent

**Figure 7:**
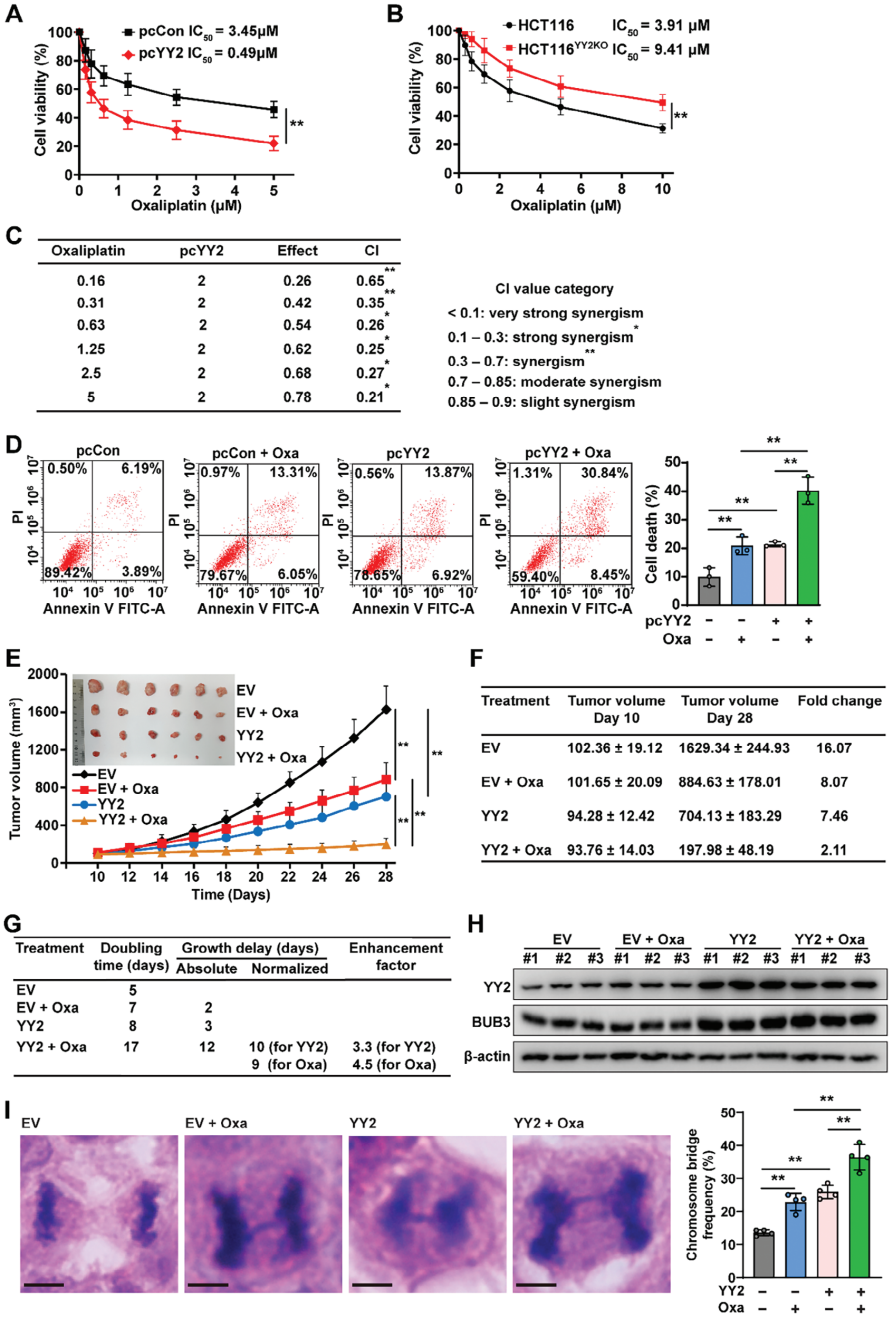
Excessive CIN sensitizes tumor cells to DNA‐damage agents. A,B) Viability of *YY2*‐overexpressed HCT116 cells (A) and HCT116^YY2KO^ cells (B) treated with indicated concentrations of oxaliplatin for 24 h. IC_50_ was calculated using CompuSyn. C) Combination index (CI) between oxaliplatin and *YY2* overexpression, as calculated using CompuSyn. D) Cell death rate of oxaliplatin‐treated *YY2*‐overexpressed HCT116 cells, as examined using Annexin V/PI staining and flow cytometry. E) Tumor volume and morphological images of xenografted tumors formed by *YY2*‐overexpressed HCT116 cells and treated with oxaliplatin at indicated time‐points (n = 6/group). F) Fold‐change of xenografted tumor volumes at day 28 compared to those at the starting point of treatment (day 10). G) Enhancement factor of combinatory treatment of oxaliplatin and *YY2* overexpression. H) YY2 and BUB3 protein levels in xenografted tumors, as determined using western blotting. I) Chromosome bridge frequency in the tissue sections of the xenografted tumors. Representative images (scale bars: 5 µm) and quantification results (each dot represents chromosome bridge frequency from one slide, with total 100 mitotic‐cells/group) are shown. β‐actin was used as western blotting loading control. Cells transfected with pcCon, wild‐type HCT116 cells, or cells infected with empty virus (EV) were used as controls. Quantification data are shown as mean ± SD. All data were obtained from three independent experiments. *P* values were calculated by one‐way ANOVA. pcCon: pcEF9‐Puro; **P < 0.01.

Given that *YY2* overexpression induces excessive CIN, we examined its synergistic effect with oxaliplatin and paclitaxel, which are DNA damage‐inducing agents that can induce CIN.^[^ ^5^, ^46^, ^47^ ^]^ *YY2* overexpression significantly decreased the IC_50_ of oxaliplatin; in contrast, *YY2* knockout robustly increased it (**Figure** **7**A,B). Similarly, *YY2* overexpression decreased the IC_50_ of paclitaxel (Figure S11A, Supporting Information). Combinatorial index (CI) analysis using a previously described method^[^ ^48^, ^49^ ^]^ showed a synergistic effect between *YY2* overexpression and oxaliplatin treatment (Figure 7C), as well as between *YY2* overexpression and paclitaxel treatment (Figure S11B, Supporting Information). The results of the EdU incorporation assay (Figure S11C,D, Supporting Information) and cell death rate (Figure 7D) showed that *YY2* overexpression further enhanced the suppressive effect of oxaliplatin on HCT116 cell proliferation potential while further promoting its effect on inducing cell death.

We then assessed the possibility of combining excessive CIN induced by *YY2* overexpression with oxaliplatin for anti‐tumor therapeutic strategy in vivo. To this end, we established a *YY2*‐overexpressed HCT116 stable cell line using a lentivirus and performed xenograft experiments (Figure S11E, Supporting Information). Whereas the volume of xenografted tumors formed by control cells increased 16‐fold within 4 weeks, *YY2* overexpression or oxaliplatin treatment alone suppressed this increase to approximately eight‐fold. The suppressive effect was further enhanced by combining *YY2* overexpression and oxaliplatin treatment, which suppressed the increase in tumor volume to only two‐fold (Figure 7E,F). The tumor morphology further confirmed this tendency (Figure 7E). Furthermore, doubling time analysis showed that combining *YY2* overexpression and oxaliplatin treatment resulted in more than 9 days of growth delay compared to that with *YY2* overexpression or oxaliplatin treatment alone, thereby enhancing the therapeutic effect by approximately four‐fold (Figure 7G). Western blotting and immunohistochemistry (IHC) staining results showed that BUB3 expression was increased in tumors formed by *YY2*‐overexpressed cells, whereas oxaliplatin did not affect YY2 and BUB3 expression (Figure 7H; Figure S11F, Supporting Information). Furthermore, IHC staining using γH2AX showed that DNA damage in tumor lesions, which was increased by *YY2* overexpression or oxaliplatin treatment alone, was further enhanced by combined oxaliplatin treatment and *YY2* overexpression (Figure S11F, Supporting Information).

Finally, we examined CIN levels in the tumor lesions. Compared to that in the controls, *YY2* overexpression or oxaliplatin treatment increased the chromosome bridge frequency, whereas in oxaliplatin‐treated *YY2*‐overexpressed HCT116 cells, this was further increased (Figure 7I). Collectively, these results demonstrated that *YY2* overexpression sensitizes tumor cells to DNA damage‐inducing agent by increasing CIN, suggesting that this combination might be a potential anti‐tumor therapeutic strategy.

Together, our results demonstrated that YY2 is a novel SAC modulator that activates *BUB3* transcription, thereby influencing SAC activity. This subsequently determines tumor cell survival and drug resistance by regulating the levels of CIN.

## 3 Discussion

Impaired SAC is the main cause of chromosome segregation errors in mitosis, leading to numerical and structural chromosome changes in tumor cells.^[^ ^12^, ^13^ ^]^ Mouse models have shown that impaired SAC promotes aneuploidy in vivo.^[^ ^19^ ^]^ Moreover, defective SAC activity is closely related to diseases such as tumors and mosaic variegated aneuploidy, a rare disorder with a high aneuploidy rate and increased tumor incidence.^[^ ^50^ ^]^ On the other hand, the role of SAC hyperactivation in tumorigenesis is still poorly understood, with conflicting reports on its pro‐ or anti‐tumor effects.^[^ ^16^, ^17^, ^39^ ^]^ Nevertheless, mutations in SAC genes are rare in human tumors, indicating that they are not major contributors to the mitotic errors observed in tumor cells.^[^ ^18^ ^]^ In this study, we found that the expression of YY2, a transcription factor highly conserved in placental mammals,^[^ ^51^, ^52^ ^]^ oscillates during the cell cycle and peaks during the M phase. Furthermore, we identified YY2 as a novel SAC modulator that directly activates the transcriptional activity of *BUB3*, a component of the SAC complex. We revealed that YY2 downregulation weakens SAC activity, thereby promoting tumorigenesis by accelerating sister chromatid segregation and mitotic exit. Meanwhile, *YY2* overexpression hyperactivates SAC, leading to prolonged mitotic time, decreased proliferation potential, and increased tumor cell death, thus suppressing tumorigenesis. It is noteworthy that even when *YY2*‐knocked out cells were treated with nocodazole, they still retained some level of SAC. This suggests that while YY2 downregulation can weaken SAC activity, it does not abolish it, further underscoring the role of YY2 as a modulator, rather than a component of the SAC. YY2 downregulation in various tumors, including colorectal, prostate, ovarian, and liver cancers, is closely associated with poor survival and prognosis.^[^ ^53^, ^54^, ^55^ ^]^ While previous studies have revealed that YY2 can trigger ferroptosis, ultraviolet damage response, and p53‐mediated cell cycle arrest,^[^ ^29^, ^31^, ^56^ ^]^ studies regarding its physiological and pathological functions are still very limited, and the mechanisms underlying its tumor suppressive effect have not been completely elucidated. Hence, our findings provide a new perspective regarding a novel function of YY2 in regulating SAC activity and subsequently, tumor cell mitotic regulation.

CIN is a hallmark of cancer and can drive tumorigenesis. Paradoxically, previous clinical studies have shown the role of CIN levels in determining tumor cell survival, in which high CIN levels before treatment are associated with improved overall survival outcomes, whereas intermediate CIN levels are associated with poor clinical outcomes.^[^ ^9^, ^10^, ^11^ ^]^ A similar phenomenon was observed in bacteria and viruses, where higher genomic instability benefits the population in stressful environments through the development of mutations that provide a selective growth advantage; however, cells with drastic instability never become dominant in a population, as their instability levels lead to deleterious mutations exceeding the viability threshold.^[^ ^57^ ^]^ In tumor cells, this concept of a “just‐right” level of instability was also observed based on mathematical modeling of the evolutionary dynamics of genetically unstable populations,^[^ ^58^, ^59^, ^60^ ^]^ suggesting that whereas moderate CIN benefits tumorigenesis as it can allow tumor cells with varying genetic alterations to have greater chance of acquiring advantageous characteristics, such as increased proliferation, excessive CIN might induce tumor cell death. Yet, experimental evidence to support this hypothesis and the molecular mechanisms underlying tumor cell CIN are still lacking.

Intriguingly, *YY2* overexpression, which hyperactivates the SAC and triggers tumor suppressive effects, also induces CIN. Indeed, previous studies have revealed that SAC hyperactivation, for example by overexpression of another SAC component, *MAD2*, or by knocking down *p31*, a component of SAC silencing, could also induce CIN and cell death.^[^ ^17^, ^39^, ^61^, ^62^ ^]^ Here we revealed that in the *YY2*‐induced SAC‐hyperactivated cell population, dying cells had significantly higher CIN than living cells, thus provides evidence that high CIN induces cell death and is thus deleterious for tumor cells. Meanwhile, although significantly lower than that in dying cells, living cells from this population also exhibited a certain degree of CIN and improved DNA‐damage resistance compared to those in control cells, suggesting that moderate CIN is beneficial for tumorigenesis. Together with the fact that knocking out *YY2* alone is not sufficient for inducing cell death, while further inhibiting SAC activity in *YY2*‐knocked out cells could also lead to excessive CIN and cell death, our findings clearly showed that different levels of CIN is crucial for determining tumor cell survival. Obviously, while excessive CIN will eventually lead to cell death, the effect of *YY2* alteration on intrinsic characteristics of the cells, such as their gene expression profiles in responses to DNA damage stress and apoptotic stimuli, also contribute greatly to their capacity in tolerating CIN stress, and subsequently, the threshold of CIN‐induced cell death. Hence, while further systematical investigation is needed to reveal the way tumor cells explore their fitness landscape upon CIN induction, our findings provide experimental evidence that conforms to the “just‐right” model.^[^ ^63^, ^64^ ^]^

DNA damage‐inducing agents such as oxaliplatin, taxol, and radiotherapy, which have been used clinically for treating tumors, can induce mitotic errors and CIN;^[^ ^5^, ^10^, ^47^, ^65^ ^]^ while SAC activator, such as MK‐1775 and ZN‐c3, are in clinical trials and are considered as potential anti‐tumor drugs.^[^ ^66^ ^]^ Meanwhile, increasing evidences suggest that a population of tumor cells remain viable after exposure to various anti‐tumor drugs. These residual cells display reduced drug sensitivity, thereby providing a reservoir of cells that might seed the growth of drug‐resistant recurrent tumor. Therefore, targeting residual cells have attracted attention as a crucial point for anti‐tumor therapeutic strategy; however, the mechanisms by which these cells are generated are still poorly understood. Our findings showed that residual tumor cells that survived DNA damage‐inducing agents as well as YY2‐induced SAC hyperactivation, possess moderate CIN and are more resistant. Hence, while the underlying mechanism needs to be further elucidated, to our knowledge, our results link up for the first time the level of CIN and residual tumor cells, thus providing new perspective regarding the generation of residual tumor cells as well as their drug resistance. Furthermore, although further study is needed, these findings also point to possible complications, such as predisposing normal or benign tumor cells to becoming more tumorigenic, owing to the induction of moderate CIN mediated by DNA damage‐based anti‐tumor therapies. Moreover, these findings indicate the possibility of applying the pretreatment CIN level as an indicator to identify patients who would benefit from DNA damage‐based anti‐tumor therapies.

Subsequently, our results also demonstrated that *YY2* overexpression‐induced SAC hyperactivation, which triggers excessive CIN, significantly sensitizes CRC cells to oxaliplatin and paclitaxel, indicating a synergism between YY2/SAC hyperactivation‐induced excessive CIN and DNA damage‐inducing agents. Combining *YY2* overexpression and oxaliplatin treatment significantly delayed tumorigenesis and enhanced the therapeutic effect in a xenograft mouse model, most plausibly by increasing CIN. Therefore, while further study is required to further elucidate whether DNA damage inducing agents, such as platinum‐based and taxol‐based antitumor drugs, could induce CIN through SAC activation, as well as the involvement of other YY2‐regulated pathways in enhancing the therapeutic effect of this combinatorial therapy, our findings suggest a potential anti‐tumor combinatory therapeutic strategy based on a DNA damage‐inducing agent and YY2‐induced excessive CIN, to improve the efficacy of the former, and at the same time, preventing the generation of residual tumor cells surviving DNA damage‐inducing agents with moderate CIN, which could trigger drug resistance.

Taken together, we identified YY2 as a novel modulator of SAC activity. Alterations in this activity can induce different degrees of CIN and influence the survival and drug response of tumor cells. This provides experimental evidence explaining the CIN paradox in cancer. Furthermore, our findings have unraveled a mechanism of drug resistance in residual cells surviving anti‐tumor therapies such as those using DNA damage‐inducing agents and SAC activation. This suggests a novel anti‐tumor therapeutic strategy that combines SAC activity regulators and DNA damage‐inducing agents.

## 4 Experimental Section

### Plasmids and Constructs

shRNA expression vectors targeting two different sites of *BUB3* were constructed using algorithm and method as described previously.^[^ ^67^ ^]^ Target sequences were predicted as follows: 5ʹ‐ GTC CAG AAG TGA ATG TGA T −3ʹ (shBUB31‐1); 5ʹ‐ AGA AGA AGA AGT ATG CCT T −3ʹ (shBUB3‐2).

*YY2* (NM_206923.4) overexpression vector (pcYY2), mutant *YY2* overexpression vectors, and lentivirus vector overexpressing *YY2* (pLenti‐YY2) were constructed as described previously.^[^ ^29^ ^]^ For *BUB3* (NM_004725.4) overexpression vectors, human complementary DNA (cDNA) obtained by reverse‐transcribing the total RNA extracted from HCT116 cells using the PrimeScript RT Reagent Kit with gDNA Eraser (Takara Bio, Dalian, China) was used as the template for amplifying the corresponding regions using the PrimeSTAR Max DNA Polymerase (Takara Bio). The amplicons were then cloned into the *Hind*III and *Eco*RI sites of pcEF9‐Puro vector.^[^ ^68^ ^]^ For FLAG‐tagged *BUB3* overexpression vectors (pcFLAG‐BUB3), the coding sequences were amplified from human cDNA as described above, and then inserted into the *Hind*III and *Eco*RI sites of pcFLAG vector as described previously.^[^ ^69^ ^]^ For doxycycline‐inducible *YY2* overexpression vector (pTRIPZ‐YY2), *YY2* coding region was inserted into *Xho*I and *Mlu*I sites of tetracycline‐inducible lentiviral pTRIPZ vector (GE Dharmacon Technology, Lafayette, CO).

For reporter vectors bringing different regions of *BUB3* promoter (Refseq No. NC_000010.11; BUB3‐Luc‐1 with the −2688 to +166 region; BUB3‐Luc‐2 with the −1765 to +166 region; BUB3‐Luc‐3 with the −1224 to +166 region; BUB3‐Luc‐4 with the −723 to +166 region; and BUB3‐Luc‐5 with the −658 to +166 region), the corresponding regions of the *BUB3* promoter were cloned into *Xho*I and *Hind*III sites of the pGL4.13 (Promega, Madison, WI). Human genome DNA extracted from HCT116 cells using the TIANamp Genomic DNA Kit (Tiangen Biotech, Beijing, China) was used as template for amplifying the promoter regions. BUB3‐luciferase vector with mutated predicted YY2 binding site (BUB3‐Luc^mut^) was constructed based on the site‐specific mutagenesis method using a Site‐directed Gene Mutagenesis Kit (Beyotime Biotechnology, Shanghai, China).

### Cell Lines and Cell Culture

HCT116 (catalog number: TCHu 99) and HEK293T (catalog number: GNHu17) cells were purchased from the Cell Bank of Chinese Academy of Sciences (Shanghai, China) and cultured in Dulbecco's modified Eagle's medium (Gibco, Life Technologies, Grand Island, NY) with 10% FBS (Biological Industries, Beith Haemek, Israel) and 1% penicillin‐streptomycin. Cell lines were verified using short‐tandem repeat profiling method and were tested periodically for mycoplasma contamination using Mycoplasma Detection Kit‐QuickTest (Biotool, Houston, TX). Transfection was performed using Lipofectamine 2000 (Invitrogen Life Technologies, Carlsbad, CA) according to the manufacturer's instruction. HCT116^YY2KO^ cell with deletion of nucleotides located in +95 to +151 region (56 bp) of *YY2* coding sequence was established using CRISPR/Cas9 method as described previously.^[^ ^29^ ^]^ For gene knockdown, overexpression, or rescue experiments, cells were seeded in 6‐well plates and transfected with 2 µg of indicated shRNA expression vectors and/or overexpression vectors. 24 h after transfection, puromycin selection (final concentration: 1 µg mL^−1^) was performed for 36 h to eliminate untransfected cells. For restoring *YY2* in *YY2*‐knocked out cells, HCT116^YY2KO^ cells were seeded in 6‐well plates and transfected with 1 µg of *YY2* overexpression vector before puromycin selection as mentioned above.

Lentiviruses for establishing *YY2*‐inducible cell lines and stable cell lines for xenograft experiments were generated by co‐transfecting HEK293T cells with 8 µg pLenti‐YY2 or pTRIPZ‐YY2 vectors, 6 µg pCMVΔR, and 2 µg pCMV‐VSVG in a 10 cm dish. Growth medium was changed the following day and lentivirus‐containing supernatant was harvested and filtered with a 0.45‐µm filter after 48 h. For infection, HCT116 cells were cultured in 6‐well plates. 24 h later, the medium was changed to 1 mL fresh culture medium and 1 mL corresponding lentivirus supernatant. Infected cells were then selected using 1 µg mL^−1^ puromycin for 7 days.

For oxaliplatin treatment, cells were seeded in 6‐well plates and cultured with medium containing indicated concentration of oxaliplatin (MedChemExpress, Monmouth Junction, NJ).

### Clinical Human Colon Carcinoma Specimen

Human colon carcinoma specimens were obtained from colon carcinoma patients undergoing surgery at Chongqing University Cancer Hospital (Chongqing, China), and stored in Biological Specimen Bank of Chongqing University Cancer Hospital. Patients did not receive chemotherapy, radiotherapy, or other adjuvant therapies prior to the surgery. The specimens were snap‐frozen in liquid nitrogen. Prior patient's written informed consents were obtained. The experiments were approved by the Institutional Research Ethics Committee of Chongqing University Cancer Hospital (Permit No. CZLS2021292‐A), and conducted in accordance with Declaration of Helsinki.

### Animal Experiments

BALB/*c‐nu*/*nu* mice (Strain number D000521, male, body weight: 18–22 g, 6 weeks‐old) were purchased from the Chongqing Medical University (Chongqing, China), randomly divided into 4 groups (n = 7), and injected subcutaneously with 3 × 10^6^ indicated stable cell lines. Oxaliplatin (MedChemExpress) was administered intraperitoneally at a dose of 5 mg kg^−1^ twice a week for 3 weeks. Treatment began on day 10, when the tumor volume reached 100 mm^3^. Tumor size (V) was evaluated by a caliper every 2 days using the following equation: V = a × b^2^/2, where a and b were the major and minor axes of the tumor, respectively. The investigator was blinded to the group allocation and during the assessment. Animal studies were approved by the Institutional Ethics Committee of Chongqing Medical University (Permit No. SYXK‐2021‐0001), and carried out in the Chongqing Medical University. All animal experiments conformed to the approved Guidelines for the Care and Use of Laboratory Animals of Chongqing Medical University. All efforts were made to minimize suffering.

### Cell Sorting

Cells were stained with Annexin V/PI according to manufacturer's instruction (Neobiosciences, Shanghai, China), and sorted with MoFlo XDP cell sorter (Beckman Coulter, Indianapolis, IN) for living (Annexin V^−^/PI^−^) and dying (Annexin^+^/PI^−^) cells. Data was analyzed using CytExpert (Beckman Coulter).

### Preparation of Cells Transiently Overexpressing YY2

*YY2*‐inducible cell line were treated with doxycycline (final concentration: 2 µg mL^−1^) for at 48 h prior to cell sorting. Following cell sorting, the collected cells were cultured in medium without doxycycline for 48 h to turn off *YY2* overexpression.

### Tumor Growth Delay and Enhancement Factor

Tumor growth delay and enhancement factor was calculated using the following equation: (1): absolute growth delay was calculated by subtracting the doubling time of the tumor in the treated group from that of the EV group; (2): normalized growth delay for YY2 group was calculated by subtracting the absolute growth delay of the YY2 + oxaliplatin group from that of the EV + oxaliplatin group; (3): normalized growth delay for oxaliplatin was calculated by subtracting the absolute growth delay of the YY2 + oxaliplatin group from that of the YY2 group; (4): enhancement factor for YY2 group was calculated by dividing the normalized growth delay for YY2 group (2) with the absolute growth delay of the YY2 group; (5): enhancement factor for oxaliplatin was calculated by dividing the normalized growth delay for oxaliplatin (3) with the absolute growth delay of the EV + oxaliplatin group.

### Live‐Cell Imaging

For fluorescence imaging of mitotic division, cells were prepared as described above prior to seeding in 3.5‐cm glass bottom dish (Wuxi NEST Biotechnology, Wuxi, China; 1 × 10^5^ cells per dish). Nuclei was stained using Hoechst 33342 (Solarbio, Beijing, China) 30 min prior to imaging. The culture was maintained at 37 °C under 5% CO_2_ in a stage‐top incubator (Tokai Hit, Shizuoka, Japan). Images were acquired every 5 min for 6 h with a 20‐ms exposure time using inverted fluorescence microscope Olympus IX83 (Olympus). Images were processed using FLUOVIEW (v.2.3). Mitotic time was quantified as the time from nuclear envelope breakdown (NEBD) until the onset of anaphase.

To study cumulative mitotic exit, nocodazole (final concentration: 100 ng mL^−1^; Beyotime Biotechnology) was added, and the cells were imaged every 10 min for 10 h using Olympus IX83 (Olympus). The number of cells that exited mitosis was quantified and analyzed over time using FLUOVIEW software (Olympus).

### RNA Sequencing and Data Analysis

RNA‐seq analysis were performed by Shanghai Bio Technology Corporation (Shanghai, China) using Illumina HiSeq 2500 (Illumina, San Diego, CA; three replicates for each group). Sequencing raw reads were pre‐processed by filtering out rRNA reads, sequencing adapters, short‐fragment reads and other low‐quality reads. Tophat v2.1.0 was used to map the cleaned reads to human reference genome ensemble GRCh38 (hg38) with two mismatches. After genome mapping, Cufflinks v2.1.1 was run with a reference annotation to generate FPKM values for known gene models. Differentially expressed genes were identified using Cuffdiff. The *P*‐value significance threshold in multiple tests was set by the false discovery rate (FDR). The fold‐changes were also estimated according to the FPKM in each sample. To identify DEGs in *YY2*‐overexpressed cells, pairwise comparisons between each transfected sample and each control sample were first performed to identify genes upregulated or downregulated in each *YY2*‐overexpressed sample with an FDR ≤ 0.05 relative to each control.

### CIN70 Scoring and Survival Data Analysis

CIN70 scores were calculated as the mean expression of the 70 probe sets matching the 70 genes of the CIN70 signature.^[^ ^41^ ^]^ The expression level of the 70 genes of CIN70 was obtained from TCGA dataset of CRC patients treated with oxaliplatin (cBioportal; coadread_tcga_pan_can_atlas_2018). All CRC samples were stratified into CIN70 quartiles according to the CIN70 scores.

### Metaphase Spread

Cells were prepared as described above and arrested at metaphase by 2 µg mL^−1^ colchicine (Aladdin, Shanghai, China) treatment for 3 h before being harvested, re‐suspended in hypotonic solution (0.075 M KCl) and incubated for 15 min at 37 °C. After cell swelling, cells were fixed using 2 mL of freshly prepared methanol‐acetic acid fixative (3:1) and collected by centrifugation. The pellet was re‐suspended in 5 mL of 3:1 methanol‐acetic acid for 30 min and dropped onto pre‐cooled slides. Images of mitotic chromosomes were acquired with fluorescence microscope (Olympus BX53, Olympus, Tokyo, Japan). Chromosome number per cell was quantified.

### Karyotype and G‐band Analysis

Cells were treated with 2 µg mL^−1^ colchicine for 1 h and collected processed as described above. After dropped onto pre‐cooled slides, the slides were allowed to dry overnight at room temperature, banded with trypsin and stained with 10% Giemsa stain (Biosharp, Shanghai, China). Images were taken using light microscopy with Olympus BX53 (Olympus) and analyzed using SmartType software (Digital Scientific, Cambridge, UK). 10 metaphase spread images were analyzed for each sample.

### Cell Cycle Enrichment and DNA Content Analysis

For cell cycle analysis, cells were prepared as described above and synchronized in G_0_ phase by FBS starvation for 24 h. Cells were washed two times in phosphate‐buffered saline (PBS) and either collected immediately (0 h) or further cultured in medium with 10% FBS for indicated time points.

For DNA content analysis, cells were prepared as described above and subjected to flow cytometry after staining the DNA with propidium iodide (PI) (NeoBiosciences). Percentage of cells with each DNA content (2N, 3N, 4N, 5–8N) were estimated using CytExpert software (Beckman Coulter). For DNA content analysis using sorted cells, cells were transfected or infected with indicated vectors and sorted for living and dying cells as described above before being stained with PI.

### Chromosome Bridge and Micronucleus Staining

Cells were prepared as described above and cultured in 3.5‐cm confocal dishes (3 × 10^4^ cells/dish) for overnight, and synchronized at prometaphase by nocodazole treatment (final concentration: 100 ng mL^−1^; Beyotime Biotechnology) for 6 h. Cells were then fixed with 4% paraformaldehyde for 30 min at room temperature and permeabilized with PBS containing 0.1% Triton X‐100 for 5 min. Nuclei were stained with DAPI (Beyotime Biotechnology) for 15 min. For chromosome bridge observation, images were taken using laser scanning confocal microscopy (Leica Microsystems TCS SP5, Heidelberg, Germany). For micronuclei observation, images were taken using fluorescence microscope (Olympus IX71, Olympus). Micronucleus rate was defined as the ratio of total micronuclei to total cell number.

For chromosome bridge observation in xenografted tumor lesions, fresh xenografted tumor lesions were fixed using 4% paraformaldehyde for overnight, embedded in paraffin, and sectioned at 4 µm thickness using a cryostat. After being dewaxed using xylene and rehydrated, sections were stained with 0.5% hematoxylin‐eosin (Sangon Bio, Shanghai, China). After samples were dehydrated and mounted in coverslip, images were taken using light microscope (Olympus BX53, Olympus).

### Cell Death Assay

Cells were prepared as described above in Materials and Methods and re‐seeded in a 6‐well plate (3 × 10^5^ cells/well). Cells were stained with Annexin V/PI (NeoBiosciences) according to manufacturer's instruction 24 h after transfection and subjected to flow cytometry. Data was analyzed using CytExpert software.

### Cell Viability Assay and Calculation of IC_50_

Cells were prepared as described above, re‐seeded into 96‐well plates (5 × 10^3^ cells/well), and treated with oxaliplatin (final concentration: 1 µM). Cell numbers were measured by colorimetric assay with MTS (Promega, Madison, WI) at the indicated time points. IC_50_ was calculated based on the results of cell viability at different dose of drugs (final concentrations: 0.16 µM, 0.31 µM, 0.63 µM, 1.25 µM, 2.5 µM, 5 µM, and 10 µM for oxaliplatin treatment; or 0.625 nM, 1.25 nM, 2.5 nM, 5 nM, 10 nM, and 20 nM for paclitaxel treatment) using Compusyn (www.combosyn.com; Combosyn Inc, Paramus, NJ). CI values were calculated using Compusyn based on corresponding cell viability assay results using the Chou‐Talalay methods (39‐40). CI values were categorized as follow: < 0.1: very strong synergism; 0.10–0.30: strong synergism; 0.30–0.70: synergism; 0.70–0.85: moderate synergism; 0.85–0.90: slight synergism; 0.90–1.10: nearly additive; 1.10–1.20: slight antagonism; 1.20–1.45: moderate antagonism; 1.45–3.30: antagonism; 3.30–10: strong antagonism; > 10: very strong antagonism.

### Preparation and Analysis of Residual Tumor Cells

*YY2*‐inducible cell line was prepared as described above and cultured without doxycycline induction. Cells were treated with oxaliplatin (final concentration: 1 µM) for 24 h prior to sorting for living cells as described above. Oxaliplatin was withdrawn after cell sorting, and living cells were further cultured in medium with or without doxycycline (final concentration: 2 µg mL^−1^) for 48 h. Cells were treated with oxaliplatin (final concentration: 1 µM) for 5 days, and cell viability was analyzed as described previously.

For micronucleus analysis, living cells were further cultured with or without doxycycline (final concentration: 2 µg mL^−1^) for 48 h after sorting. Micronucleus staining was performed as described above in Materials and Methods.

### 5‐ethynyl‐2′‐deoxyuridine (EdU) Incorporation Assay and Colony‐formation Assay

Cells were prepared as described above and then re‐seeded in 48‐well plate (5 × 10^4^ cells/well). EdU incorporation and staining were performed using BeyoClick EdU Cell Proliferation Kit with Alexa Fluor 488 (Beyotime Biotechnology) according to the manufacturer's instruction. Nuclei were stained with Hoechst. Images were taken with fluorescence microscope (Olympus IX71). Quantification of EdU‐positive and Hoechst‐positive cells were shown as the ratio of EdU‐positive cells to Hoechst‐positive cells.

For colony‐formation assay, 300 cells were cultured in a 6‐well plate for 10 days. Cells were then fixed with 4% paraformaldehyde and stained with methylene blue. The colonies were then counted. The investigator was blinded during the assessment.

### Dual Luciferase Reporter Assay

Cells were seeded into 24‐well plates (8 × 10^4^ cells/well). Twenty‐four hours later cells were co‐transfected with the indicated overexpression vector, reporter vector, and *Renilla* luciferase expression vector (pRL‐SV40, Promega) as the internal control. Luciferase activities were measured using Dual Luciferase Assay System (Promega) 48 h after transfection. Firefly luciferase activities were normalized with the corresponding *Renilla* luciferase activities.

### Chromatin Immunoprecipitation (ChIP) Assay

Chromatin was immunoprecipitated using ChIP Assay Kit (Beyotime Biotechnology) according to the manufacturer's instructions. Briefly, cells were lysed, and chromatin was immunoprecipitated using protein A + G agarose/salmon sperm DNA and anti‐YY2 antibody or normal rabbit IgG, uncrosslinked for 4 h at 65 °C, and treated with 0.5 M EDTA, 1 M Tris (pH 6.5) and 20 mg mL^−1^ proteinase K. Immunoprecipitated chromatin was then subjected to PCR with PrimeSTAR Max (Takara Bio). Primer sequences for amplifying *BUB3* promoter region containing the predicted YY2‐binding site were 5′‐GCTGTCGTTTCAGGACCCTT‐3′ (forward primer) and 5′‐AATCAGACCAGCCTTTGCCC‐3′ (reverse primer).

### RNA Extraction and Quantitative Real‐Time PCR (qRT‐PCR)

Total RNA was extracted using TRIzol (Invitrogen Life Technology, Carlsbad, CA) according to the manufacturer's instructions. Total RNA (1 µg) was then reverse transcribed into cDNA using a PrimeScript Reagent Kit with gDNA Eraser (Takara Bio). qRT‐PCR was performed using SYBR Premix Ex Taq (Takara Bio). The sequences of the primers used were listed in Table S1 (Supporting Information). β‐actin was used to normalize sample amplifications.

For absolute qRT‐PCR, purified PCR amplicons with concentrations ranging from 1 × 10^1^ to 1 × 10^7^ copies mL^−1^ were used as standards. Briefly, amplicons were purified by gel electrophoresis followed by purification using Universal DNA Purification Kit (Tiangen Biotech). The concentration of the purified amplicons were measured using NanoDrop (Thermo Scientific, Waltham, MA). Concentration of the DNA copies for each amplicons were obtained using the following equation: DNA (copies/µl) = 6.02 × 10^23^ (copies mol^−1^) × DNA concentration (g µl^−1^) / [DNA length (bp) × 660 (g mol^−1^ bp^−1^)].

### Western Blotting

Total cells were lysed with RIPA lysis buffer supplemented with a protease inhibitor and phosphatase inhibitor cocktail (complete cocktail, Roche Applied Science, Mannheim, Germany). For samples from xenografted tumors, frozen specimens were homogenized with RIPA lysis buffer with protease inhibitor and phosphatase inhibitor cocktail to obtain protein extracts. Samples with equal amounts protein were electrophoresed on sodium dodecyl sulfate‐polyacrylamide gels before being transferred to a polyvinylidene fluoride membrane with 0.45‐µm pore size (Millipore, Billerica, MA). Antibodies used were listed in Table S2 (Supporting Information), and immunoblotting with an anti‐β‐actin antibody was conducted to ensure equal protein loading. Signals were detected using the SuperSignal West Femto Maximum Sensitivity Substrate detection system (Thermo Scientific).

### Protein Degradation Assay

For protein degradation assay, cells were synchronized at prometaphase by nocodazole treatment, as described above in Materials and Methods. Mitotic cells were washed two times in PBS and either collected immediately (0 h) or released in drug‐free medium. Protein samples were collected at the indicated time points and were subjected to western blotting as described above. Protein half‐life was determined by quantifying western blotting results.

### Immunohistochemistry and Hematoxylin‐Eosin Staining

Fresh xenografted tumor lesions were fixed using 4% paraformaldehyde for overnight, embedded in paraffin, and sectioned at 4 µm thickness using a cryostat. After being dewaxed using xylene and rehydrated, sections were subjected to immunohistochemical staining. Briefly, the tissue sections were incubated with primary antibodies for 1 h. The specimens were then incubated with corresponding second antibodies conjugated with horse‐radish peroxidase. Visualization was performed using a DAB Kit (DAKO, Beijing, China) under microscope. The nuclei were then counterstained with hematoxylin (Beyotime Biotechnology), then the sections were dehydrated and mounted with coverslip. Antibodies used were listed in Table S2 (Supporting Information). Images were taken by using Pannoramic Midi (3DHistech, Budapest, Hungary).

### Statistical Analysis

β‐actin was used for normalization in qRT‐PCR experiment and in quantifying protein expression levels for calculation of protein half‐life. For dual luciferase reporter assay, firefly luciferase activities were normalized with those of *Renilla*. All values of the experimental results were presented as mean ± SD (n = 3; unless further indicated). Quantification data were analyzed by one‐way ANOVA conducted using SPSS Statistics v20.0 (IBM, Chicago, IL). A value of *P* < 0.05 was considered statistically significant.

## Conflict of Interest

The authors declare no conflict of interest.

## Author Contributions

V.K. and S.W. conceptualized and supervised this study; R.H., I.T.S.M., W.D., and W.L. performed the experiments; R.H., I.T.S.M., W.D., M.W., S.H., M.M., V.K., and S.W. analyzed and interpreted the data; R.H., W.D., V.K., and S.W. wrote the paper; H.Z. collected human clinical samples and performed clinical samples analysis; M.M. predicted and constructed shRNA expression vectors.

## Supporting information

Supporting Information

Supplementary Video S1

Supplementary Video S2

Supplementary Video S3

Supplementary Video S4

Supplementary Video S5

Supplementary Video S6

Supplementary Video S7

Supplementary Video S8

Supplementary Video S9

Supplementary Video S10

## Data Availability

The data that support the findings of this study are available from the corresponding author upon reasonable request.
