# Supplementary material for: YY2/BUB3 Axis promotes SAC Hyperactivation and Inhibits Colorectal Cancer Progression via Regulating Chromosomal Instability
Source: Adv Sci (Weinh). 2024 Apr 29;11(26):2308690. doi: 10.1002/advs.202308690 (PMC11234461; doi:10.1002/advs.202308690)
Supplement: Supplementary file 1 — Supporting Information [file ADVS-11-2308690-s008.pdf]

ADVANCED SCIENCE
Open Access

Supporting Information

for *Adv. Sci.*, DOI 10.1002/advs.202308690

YY2/BUB3 Axis promotes SAC Hyperactivation and Inhibits Colorectal Cancer Progression via Regulating Chromosomal Instability

Rendy Hosea, Wei Duan, Ian Timothy Sembiring Meliala, Wenfang Li, Mankun Wei, Sharon Hillary, Hezhao Zhao, Makoto Miyagishi, Shourong Wu and Vivi Kasim**

Supporting Information for

YY2/BUB3 axis promotes SAC hyperactivation and inhibits colorectal cancer progression via regulating chromosomal instability

Rendy Hosea, Wei Duan, Ian Timothy Sembiring Meliala, Wenfang Li, Mankun Wei, Sharon Hillary, Hezhao Zhao, Makoto Miyagishi, Shourong Wu*, Vivi Kasim*

*E-mail: shourongwu@cqu.edu.cn
vivikasim@cqu.edu.cn

This PDF file includes:

Figure S1. YY2 regulates M phase progression.

Figure S2. YY2 induces degradation of cyclin B and securin proteins.

Figure S3. SAC is crucial for YY2 tumor suppressor activity.

Figure S4. YY2 enhances *BUB3* mRNA expression levels.

Figure S5. YY2 regulates mitosis and tumorigenic potential through BUB3-induced SAC hyperactivation.

Figure S6. Alteration in YY2 expression induces CIN.

Figure S7. *BUB3* overexpression induces CIN.

Figure S8. *YY2* overexpression induces CIN by hyperactivating SAC.

Figure S9. SAC inhibition induces excessive CIN in *YY2*-knockout cells.

Figure S10. *YY2*-transient overexpression induces heritable CIN.

Figure S11. YY2 enhances DNA-damage inducing agent tumor suppressive activity.

Figure S12. Uncropped western blots with the indicated areas of selection in Figs 1, 3, 7, and Supplementary Figs S1, S2, S3, S4, S5, S9, S10, and S11.

Table S1. Primer pairs used for qRT-PCR.

Table S2. Antibodies used for western blotting, immunohistochemistry, and ChIP assay.

Video Legends S1-S10

Other Supplementary Materials for this manuscript include the following:

Videos S1 to S10

Supplementary Figure 1

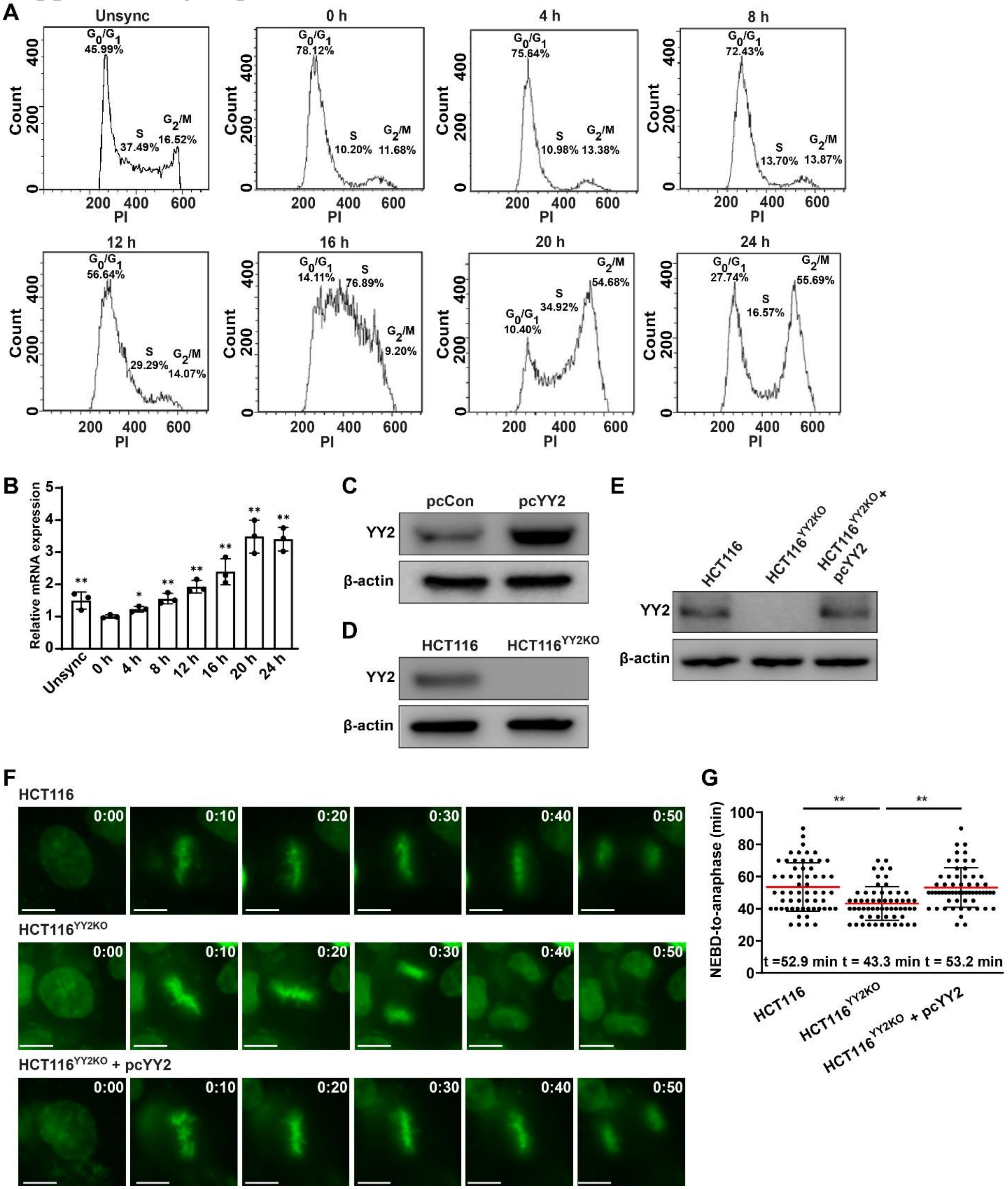

Figure S1. YY2 regulates M phase progression. A) Analysis of cell cycle progression at indicated time-points starting immediately after serum starvation release, as examined using PI staining and flow cytometry. Representative images are shown. B) YY2 mRNA expression level in HCT116 cells at indicated time-points after serum starvation release, as analyzed using qRT-PCR. YY2 mRNA levels in each time-point were shown as relative to that at 0 h after serum starvation release,

which was assumed as 1. C–D) YY2 protein expression level in HCT116 cells transfected with 2 μg *YY2* overexpression vector (C) and in $HCT116^{YY2KO}$ cells (D), as determined using western blotting. E) YY2 protein expression level in $HCT116^{YY2KO}$ cells transfected with 1 μg *YY2* overexpression vector. F–G) Mitotic time of *YY2* reintroduced-$HCT116^{YY2KO}$ cells, as determined using time-lapse microscopy. Representative images (F; scale bars: 20 μm) and scatter plot showing the time-length from NEBD to anaphase (G; total n = 60, pooled from three independent experiments) are shown. Cells transfected with pcCon or wild-type HCT116 cells were used as controls. β-actin was used for qRT-PCR normalization and as western blotting loading control. Quantification data are shown as mean ± SD. All data were obtained from three independent experiments. *P* values were calculated by one-way ANOVA. Unsync: unsynchronized cells; pcCon: pcEF9-Puro; * $P < 0.05$; ** $P < 0.01$.

Supplementary Figure 2

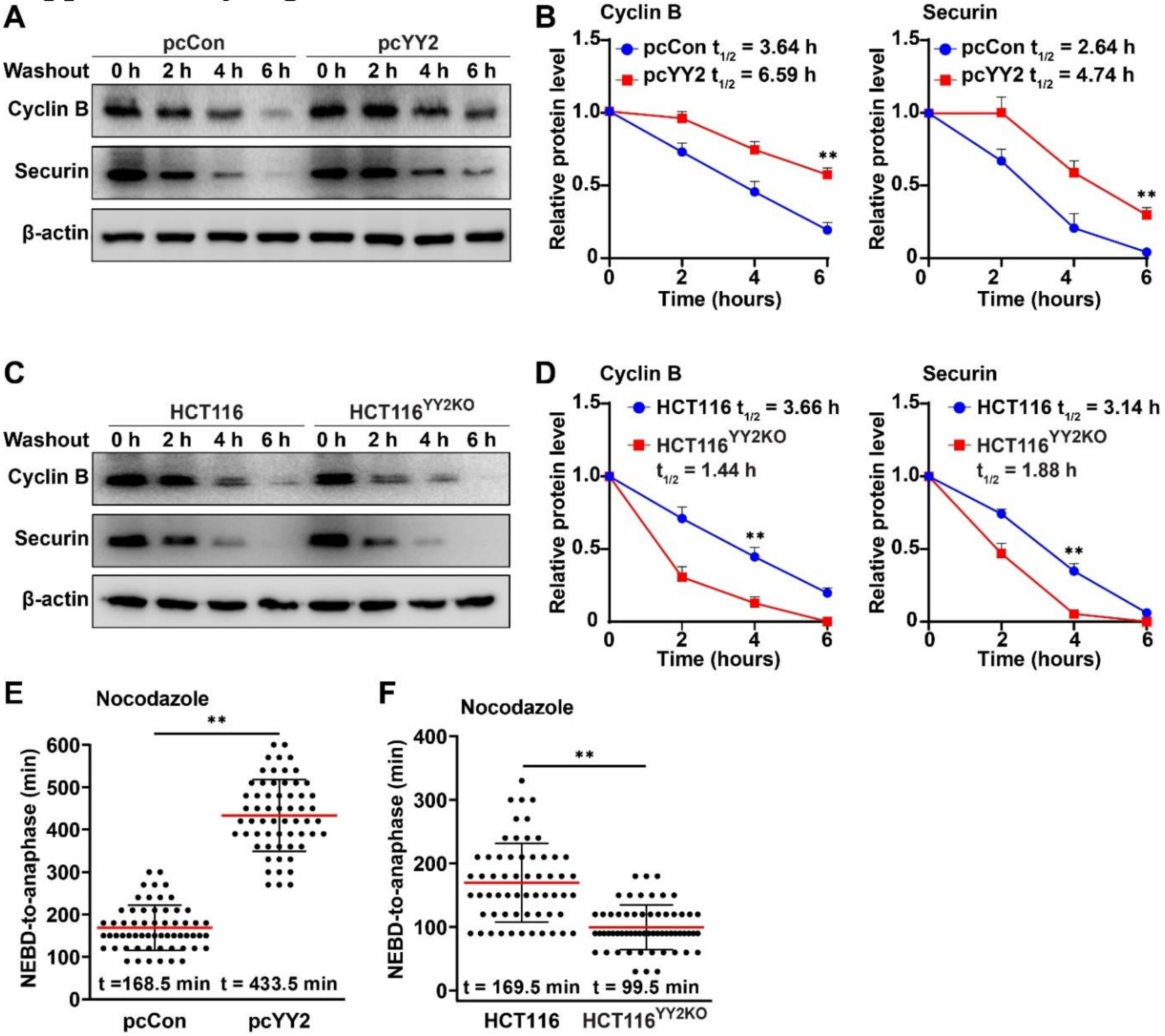

Figure S2. YY2 induces degradation of cyclin B and securin proteins. A–B) Cyclin B and securin protein expression levels in *YY2*-overexpressed HCT116 cells at indicated time-points after nocodazole washout, as analyzed using western blotting. Representative images (A) and the quantification results (B) are shown. C–D) Cyclin B and securin protein expression levels in HCT116^{YY2KO} cells at indicated time-points after nocodazole washout, as analyzed using western blotting. Representative images (C) as well as quantification results (D) are shown. E–F) Scatter plots showing the time-length from NEBD to anaphase of nocodazole-treated *YY2*-overexpressed HCT116 cells (E) and nocodazole-treated HCT116^{YY2KO} cells (F) nocodazole final concentration: 50 ng mL^{-1}; total n = 60, pooled from three independent experiments). Cells transfected with pcCon or wild-type HCT116 cells were used as controls. β-actin was used for western blotting loading control. Quantification data are shown as mean ± SD. All data were obtained from three independent experiments. *P* values were calculated by one-way ANOVA. pcCon: pcEF9-Puro; $^{**}P < 0.01$.

Supplementary Figure 3

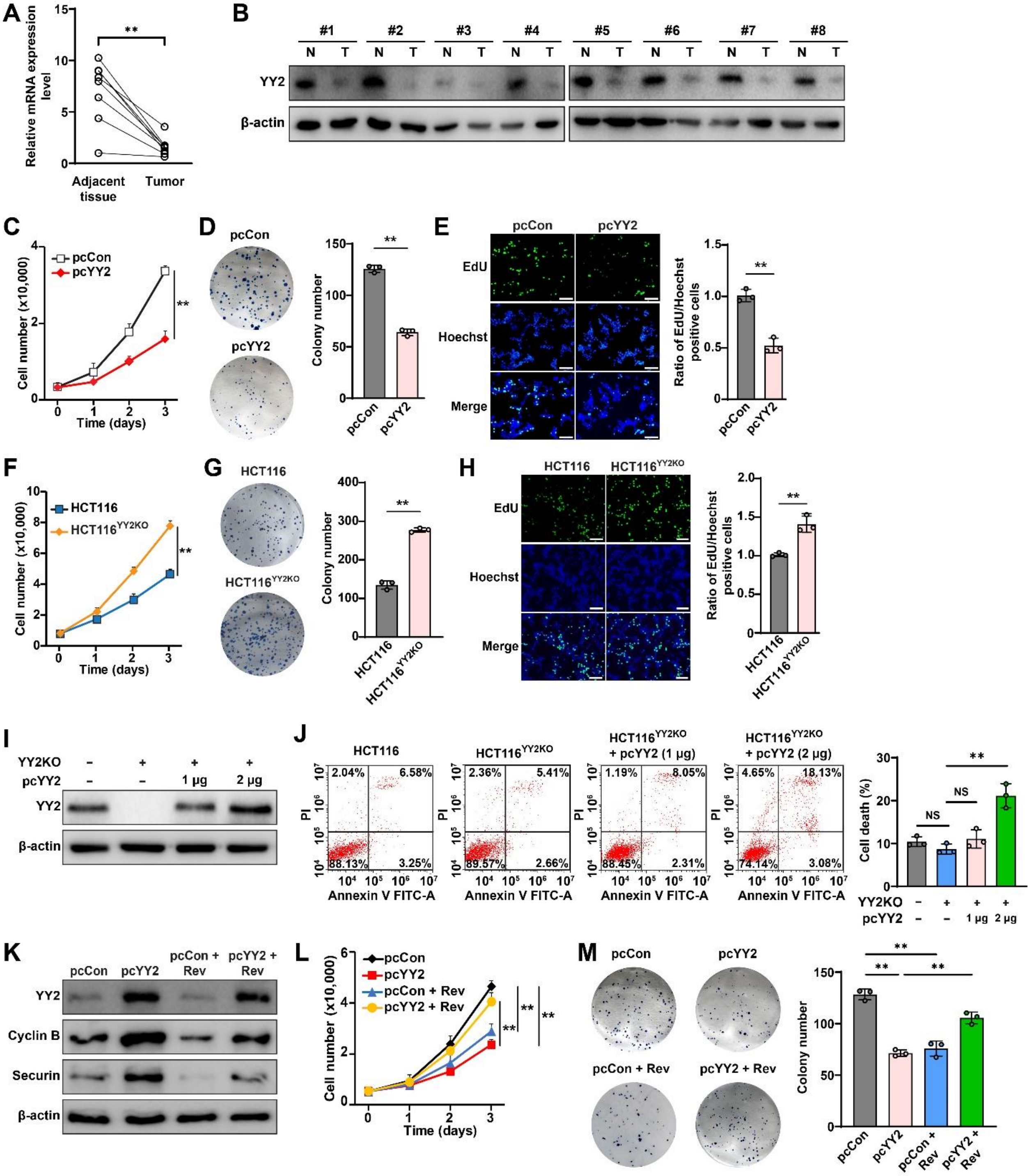

Figure S3. SAC is crucial for YY2 tumor suppressor activity. A–B) YY2 mRNA (A; n = 16) and protein (B; n = 8) expression levels in clinical human CRC and corresponding normal adjacent tissues, as analyzed using qRT-PCR and western blotting, respectively. C) Viability of *YY2*-overexpressed HCT116 cells at indicated time-points. D) Colony-formation potential of *YY2*-overexpressed HCT116 cells. Representative images (left) and colony numbers (right; each dot represents the mean value of three technical replicates) are shown. E) Proliferation potential of

YY2-overexpressed HCT116 cells, as determined using EdU-incorporation assay. Representative images (left; scale bars: 200 μm) and ratio of proliferative cells (right; each dot represents the mean value of three technical replicates) are shown. F) Viability of HCT116^{YY2KO} cells at indicated time-points. G–H) Colony-formation (G) and proliferation (H) potentials of HCT116^{YY2KO} cells. I) YY2 protein expression level in *YY2*-restored HCT116^{YY2KO} cells, as determined using western blotting. J) Cell death rate of *YY2*-restored HCT116^{YY2KO} cells, as examined using Annexin V/PI staining. K) Cyclin B and securin protein expression levels in HCT116 cells overexpressing *YY2* and treated with reversine, as determined using western blotting. L) Viability of HCT116 cells overexpressing *YY2* and treated with reversine at indicated time points. M) Colony-formation potential of HCT116 cells overexpressing *YY2* and treated with reversine. Cells transfected with pcCon or wild-type HCT116 cells were used as controls. β-actin was used for qRT-PCR normalization and as western blotting loading control. Quantification data are shown as mean ± SD. All data were obtained from three independent experiments. *P* values were calculated by one-way ANOVA. pcCon: pcEF9-Puro; Rev: reversine (final concentration: 0.2 μM); $^{**}P < 0.01$.

Supplementary Figure 4

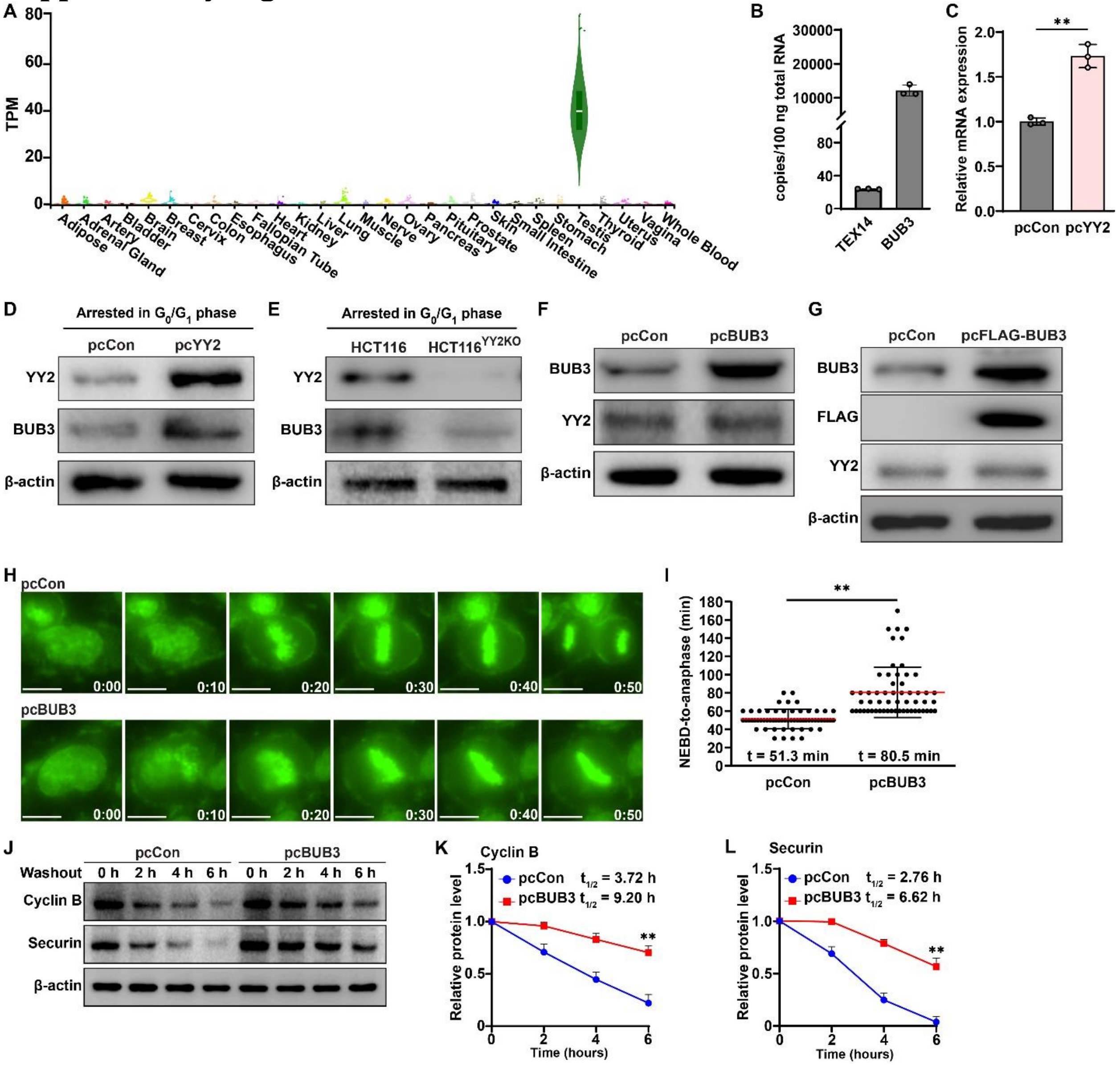

Figure S4. YY2 enhances *BUB3* mRNA expression levels. A) GTEx tissue-wide expression profiles of TEX14. B) Copy numbers of TEX14 and BUB3 mRNA in HCT116 cells, as analyzed using absolute qRT-PCR. C) BUB3 mRNA expression level in HCT116 cells overexpressing *YY2*, as analyzed using qRT-PCR. D–E) BUB3 protein expression level in *YY2*-overexpressed (D) and *YY2*-knockout (E) HCT116 cells arrested at G_0/G_1 phase following continuous serum starvation treatment, as determined using western blotting. F–G) BUB3 protein expression level in HCT116 cells transfected with *BUB3* overexpression vector (F) or *FLAG-BUB3* overexpression vector (G), as determined by western blotting using anti-BUB3 or anti-FLAG antibody, respectively. H–I) Mitotic time of *BUB3*-overexpressed HCT116 cells, as determined using time-lapse microscopy. Representative images (H; scale bars: 20 μm) and scatter plot showing the time-length from NEBD to anaphase (I; total n = 60, pooled from three independent experiments). J–L) Cyclin B and securin

protein expression levels in *BUB3*-overexpressed HCT116 cells at indicated time-points after nocodazole washout, as analyzed using western blotting. Representative images (J) as well as quantification results of cyclin B (K) and securin (L) are shown. Cells transfected with pcCon or wild-type HCT116 cells were used as control. β-actin was used for qRT-PCR normalization and as western blotting loading control. Quantification data are shown as mean ± SD. All data were obtained from three independent experiments. *P* values were calculated by one-way ANOVA. pcCon: pcEF9-Puro; ***P* < 0.01.

Supplementary Figure 5

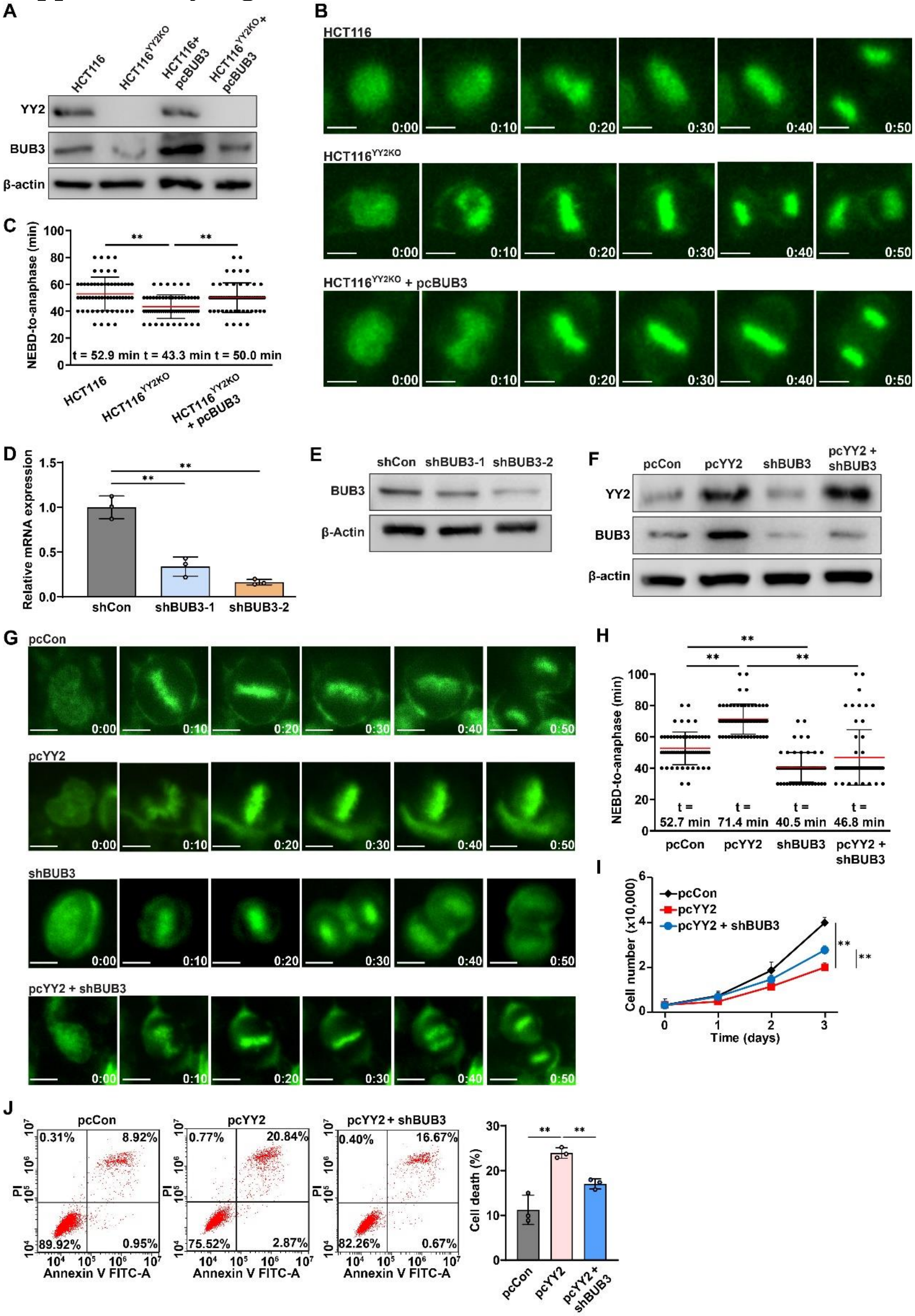

Figure S5. YY2 regulates mitosis and tumorigenic potential through BUB3-induced SAC hyperactivation. A) BUB3 and YY2 protein expression levels in *BUB3*-overexpressed

HCT116^{YY2KO} cells, as determined using western blotting. B–C) Mitotic time of *BUB3*-overexpressed HCT116^{YY2KO} cells, as determined using time-lapse microscopy. Representative images (B; scale bars: 20 μm) and scatter plot showing the time-length from NEBD to anaphase (C; total n = 60, pooled from three independent experiments) are shown. D–E) BUB3 mRNA (D) and protein (E) expression levels in HCT116 cells transfected with shRNA expression vectors targeting different sites of *BUB3*, as analyzed using qRT-PCR and western blotting, respectively. F) BUB3 and YY2 protein expression levels in *BUB3* knocked-down, *YY2*-overexpressed HCT116 cells, as determined using western blotting. G–H) Mitotic time of *BUB3* knocked-down, *YY2*-overexpressed HCT116 cells, as determined using time-lapse microscopy. Representative images (G; scale bars: 20 μm) and scatter plot showing the time-length from NEBD to anaphase (H; total n = 60, pooled from three independent experiments). I–J) Viability (I) and cell death rate (J) of *BUB3* knocked-down, *YY2*-overexpressed HCT116 cells at indicated time points. Cells transfected with pcCon, shCon, or both pcCon and shCon were used as controls. β-actin was used for qRT-PCR normalization and as western blotting loading control. Quantification data are shown as mean ± SD. All data were obtained from three independent experiments. *P* values were calculated by one-way ANOVA. pcCon: pcEF9-Puro. $^{**}P < 0.01$.

Supplementary Figure 6

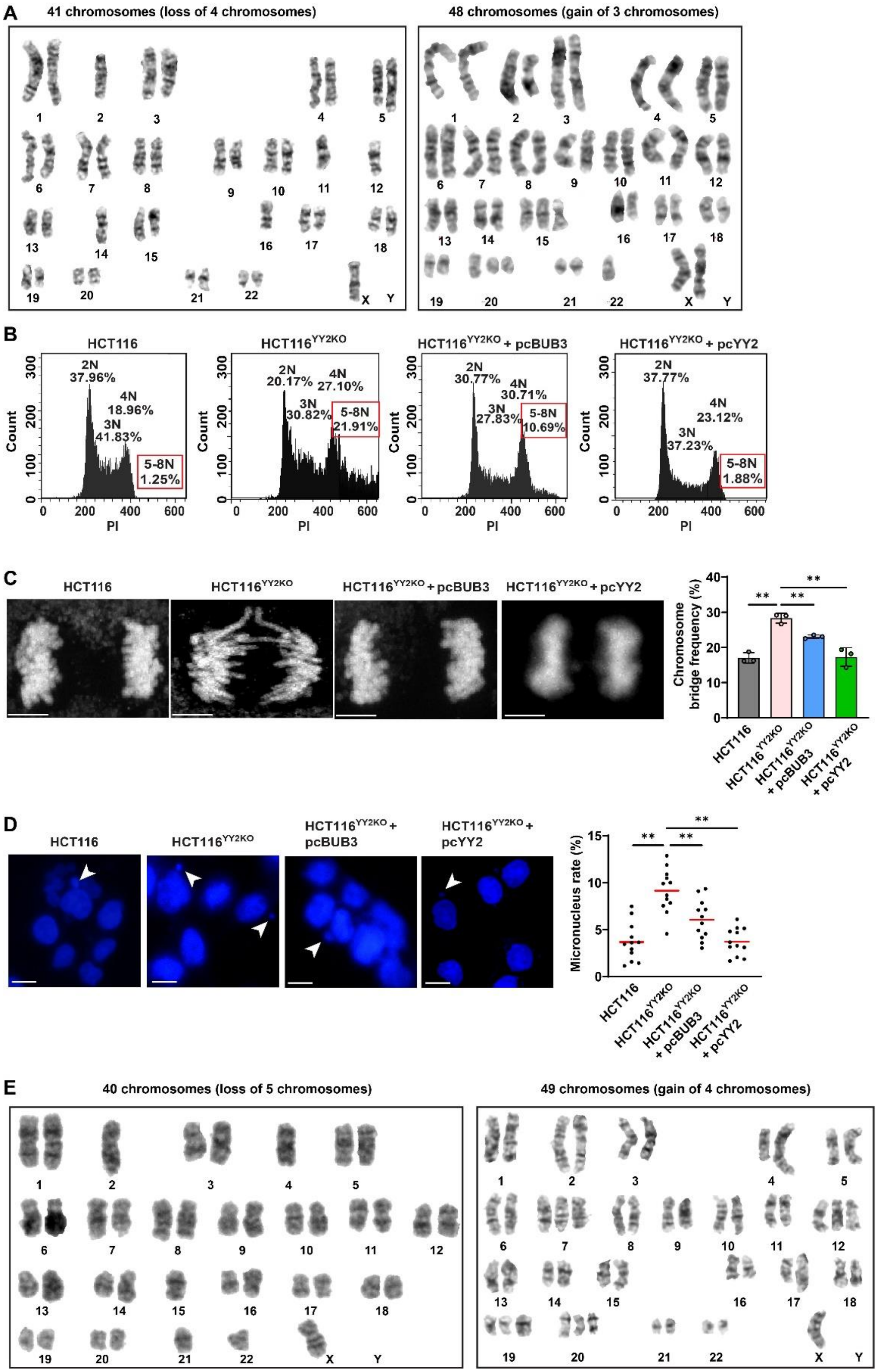

Figure S6. Alteration in YY2 expression induces CIN. A) Single karyotype analysis of HCT116^{YY2KO} cells. Representative images indicating chromosomes gain or chromosomes losses

are shown. B) DNA content in *BUB3*-overexpressed HCT116^{YY2KO} cells, as examined using PI staining and flow cytometry. C) Chromosome bridge frequency in *BUB3*-overexpressed HCT116^{YY2KO} cells. Representative images (scale bars: 5 μm) and quantification results (each dot represents chromosome bridge frequency from one independent experiment, with total 100 mitotic-cells/group) are shown. D) Micronucleus rate in *BUB3*-overexpressed HCT116^{YY2KO} cells. Representative images of micronuclei (indicated by arrowheads; scale bars: 20 μm) and micronucleus rate (ratio of micronuclei number to total cell number; each dot represents micronucleus rate/slide with > 100 cells/slides; four technical replicates from three independent experiments) are shown. E) Single karyotype analysis of *YY2*-overexpressed HCT116 cells. Representative images indicating chromosomes gain or chromosomes losses are shown. Wild-type HCT116 cells were used as controls. Quantification data are shown as mean ± SD of three independent experiments. *P* values were calculated by one-way ANOVA. $^{**}P < 0.01$.

Supplementary Figure 7

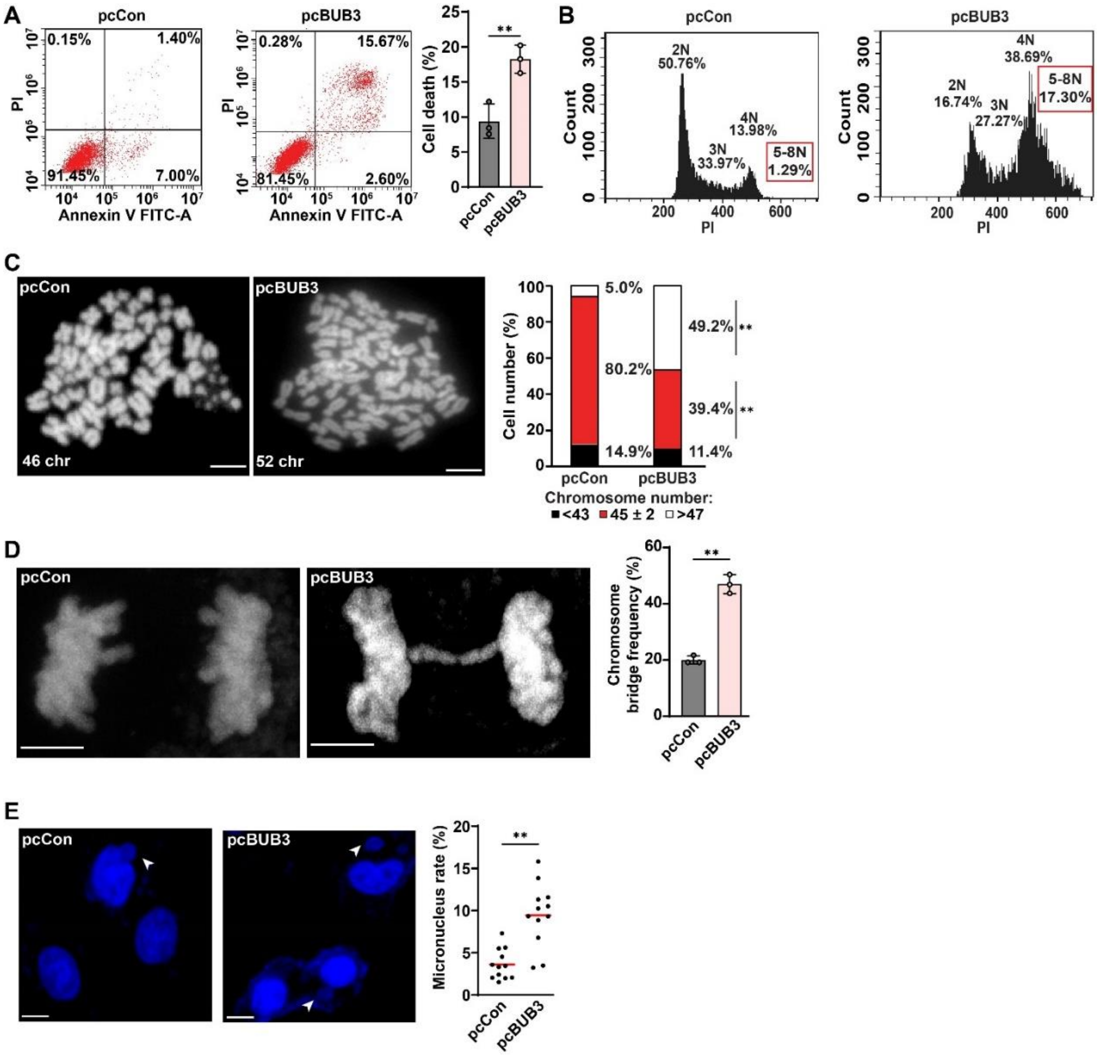

Figure S7. *BUB3* overexpression induces CIN. A) Cell death rate of *BUB3*-overexpressed HCT116 cells, as examined using Annexin V/PI staining and flow cytometry. B) DNA content in *BUB3*-overexpressed HCT116 cells, as examined using PI staining and flow cytometry. C) Chromosome number per cell in *BUB3*-overexpressed HCT116 cells, as analyzed using metaphase spread. Representative images (scale bars: 10 μm) and percentage of cells with indicated chromosome number (total cells counted: 50 cells/group, pooled from three independent experiments) are shown. D) Chromosome bridge frequency in *BUB3*-overexpressed HCT116 cells. Representative images (scale bars: 5 μm) and quantification results (each dot represents chromosome bridge frequency from one independent experiment, with total 100 mitotic-cells/group) are shown. E) Micronucleus rate in *BUB3*-overexpressed HCT116 cells. Representative images of micronuclei (indicated by arrowheads; scale bars: 20 μm) and micronucleus rate (ratio of micronuclei number to total cell number; each dot represents micronucleus rate/slide with > 100 cells/slides; four technical replicates from three independent experiments) are shown. Cells transfected with pcCon were used as controls. Quantification data are shown as mean ± SD of three independent experiments. *P* values were calculated by one-way ANOVA. pcCon: pcEF9-Puro; ***P* < 0.01.

Supplementary Figure 8

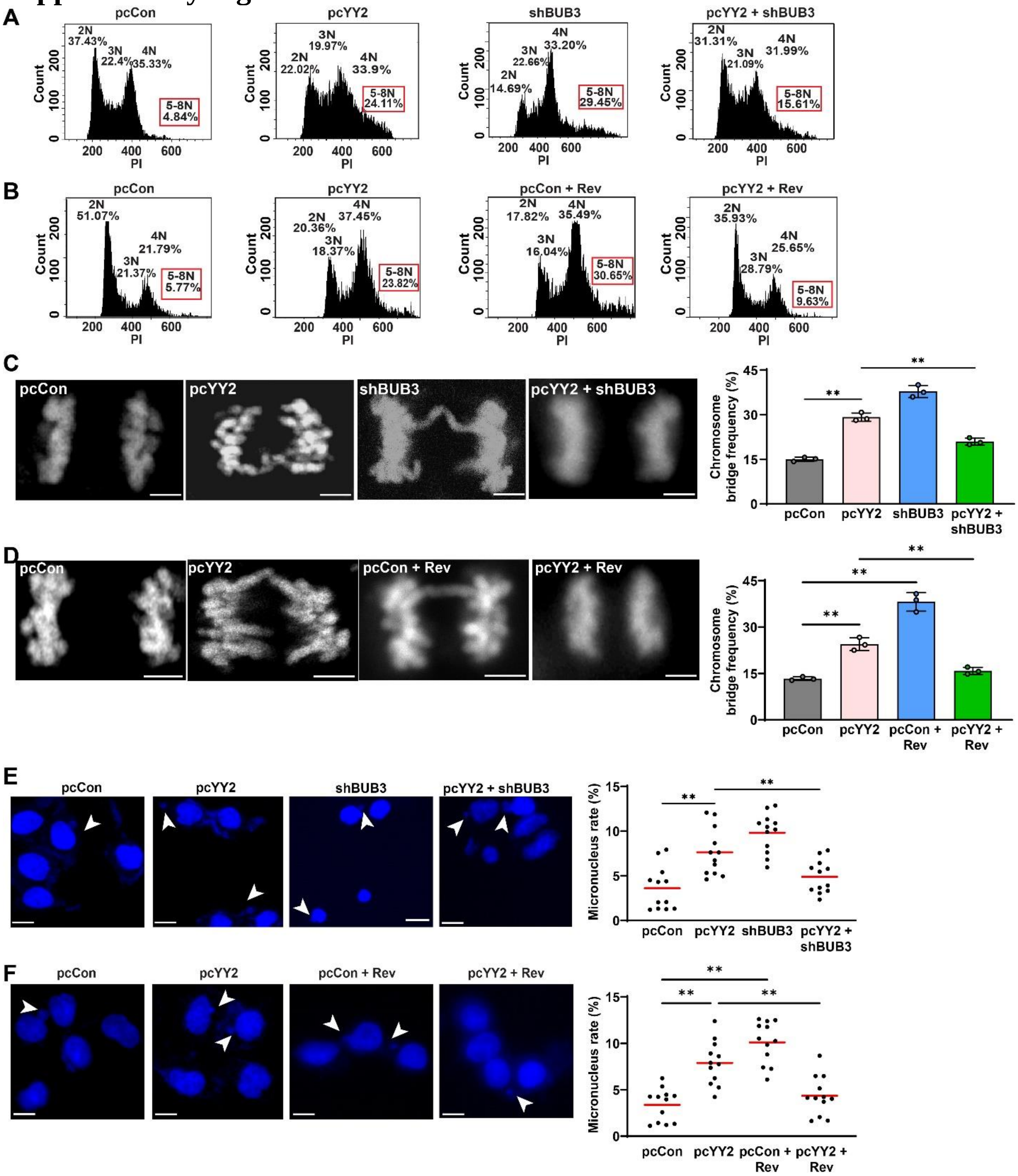

Figure S8. *YY2* overexpression induces CIN by hyperactivating SAC. A–B) DNA content in *BUB3*-knocked down, *YY2*-overexpressed HCT116 cells (A) and *YY2*-overexpressed, reversine-treated HCT116 cells (B), as examined using PI staining and flow cytometry. C–D) Chromosome bridge frequency in *BUB3*-knocked down, *YY2*-overexpressed HCT116 cells (C) and *YY2*-overexpressed, reversine-treated HCT116 cells (D), as stained using DAPI. Representative images (scale bars: 5 μm) and quantification results (each dot represents chromosome bridge frequency

from one independent experiment, with total 100 mitotic-cells/group) are shown. E–F) Micronucleus rate in *BUB3*-knocked down, *YY2*-overexpressed HCT116 cells (E) and *YY2*-overexpressed, reversine-treated HCT116 cells (F), as stained using DAPI. Representative images of micronuclei (indicated by arrowheads; scale bars: 20 μm) and quantification results (ratio of micronuclei number to total cell number; each dot represents micronucleus rate/slide with > 100 cells/slides; four technical replicates from three independent experiments) are shown. Cells transfected with pcCon, or pcCon and shCon, were used as controls. Quantification data are shown as mean ± SD. All data were obtained from three independent experiments. *P* values were calculated by one-way ANOVA. pcCon: pcEF9-Puro. Rev: reversine (final concentration: 0.2 μM). $^{**}P < 0.01$.

Supplementary Figure 9

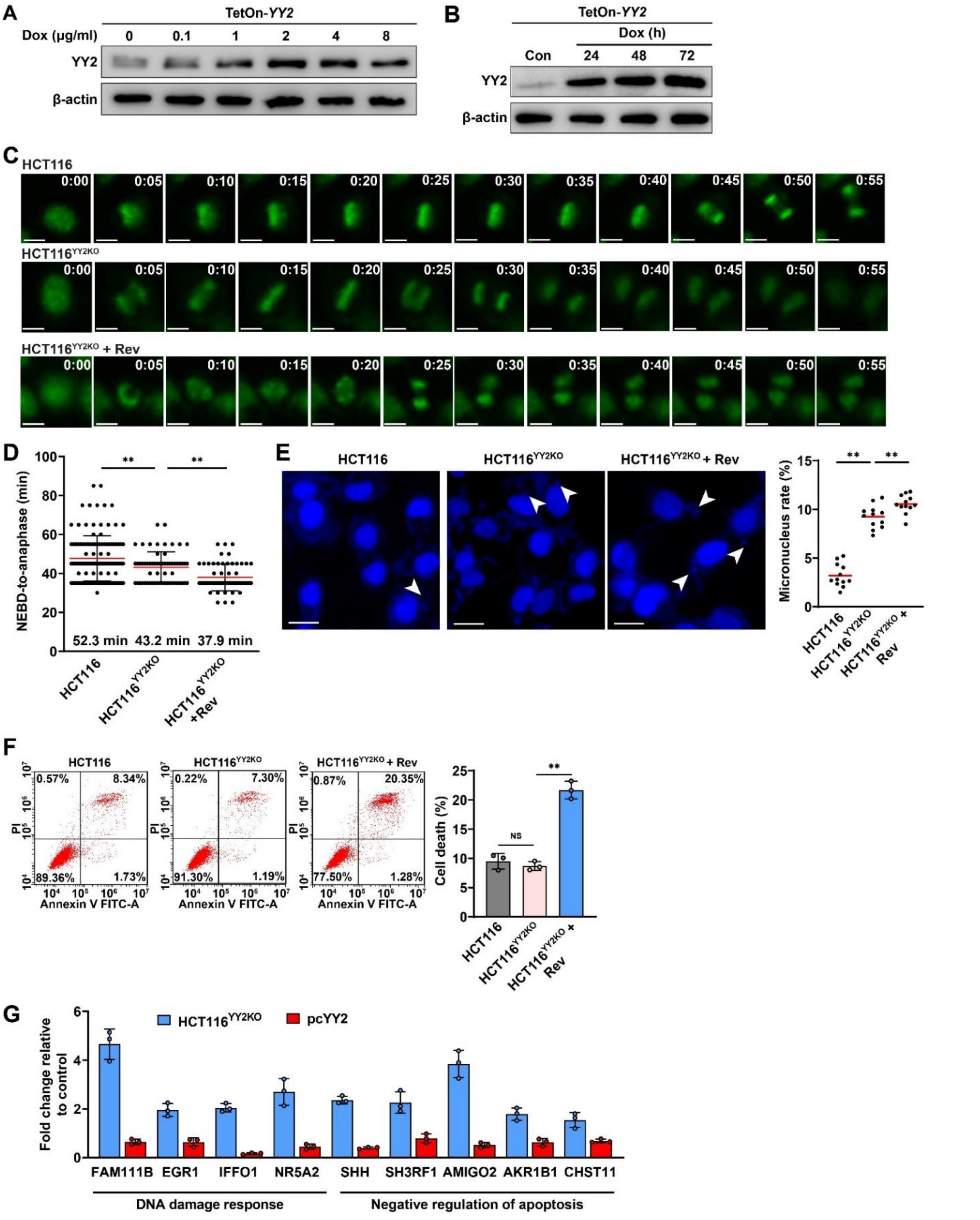

Figure S9. SAC inhibition induces excessive CIN in *YY2*-knockout cells. A–B) YY2 protein expression level in HCT116 cells infected with TetOn-*YY2* lentivirus after treatment with indicated

dose of doxycycline for 48 h (A) or with 2 μg mL^{-1} doxycycline for indicated time (B), as determined using western blotting. C–D) Mitotic time of reversine-treated $HCT116^{YY2KO}$ cells, as determined using time-lapse microscopy. Representative images (C; scale bars: 20 μm) and scatter plot showing the time-length from NEBD to anaphase (D; total n = 60, pooled from three independent experiments) are shown. E) Micronucleus rate in reversine-treated $HCT116^{YY2KO}$ cells, as stained using DAPI. Representative images of micronuclei (indicated by arrowheads; scale bars: 20 μm) and quantification results (ratio of micronuclei number to total cell number; each dot represents micronucleus rate/slide with >100 cells/slides; four technical replicates from three independent experiments) are shown. F) Cell death rate of reversine-treated $HCT116^{YY2KO}$ cells, as examined using Annexin V/PI staining. G) Fold change of mRNA expression levels of genes related to DNA damage response and negative regulation of apoptosis in $HCT116^{YY2KO}$ cells compared to wild-type HCT116 cells, and in *YY2*-overexpressed HCT116 cells compared to HCT116 cells transfected with pcCon, as analyzed using qRT-PCR. β-actin was used for qRT-PCR normalization and as western blotting loading control. Quantification data are shown as mean ± SD. All data were obtained from three independent experiments. *P* values were calculated by one-way ANOVA. Rev: Reversine (final concentration: 0.2 μM); $^{**}P < 0.01$; NS: not significant.

Supplementary Figure 10

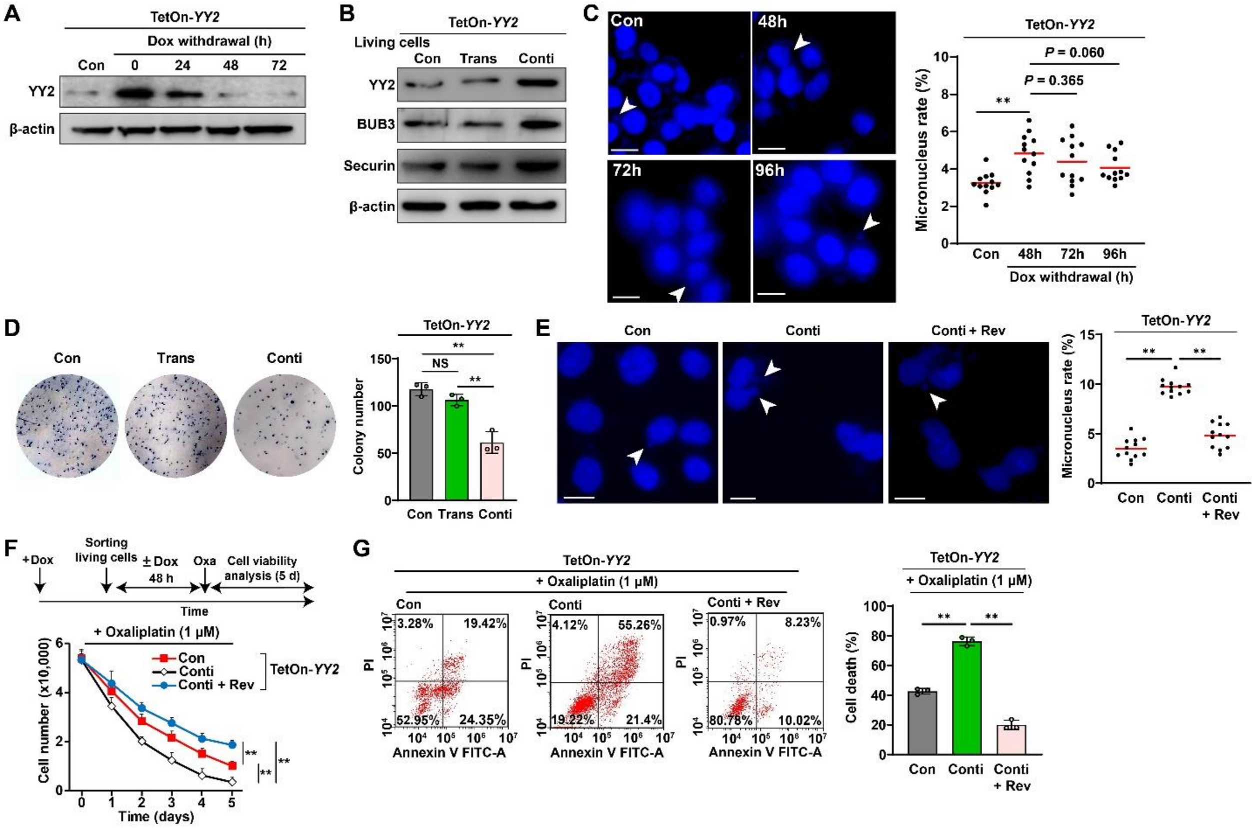

Figure S10. *YY2*-transient overexpression induces heritable CIN. A) YY2 protein expression level in HCT116 cells transiently overexpressing *YY2* at indicated time-points after doxycycline-withdrawal, as determined using western blotting. B) YY2, BUB3, and securin protein expression levels in HCT116 cells transiently overexpressing *YY2* (TetOn-*YY2* Trans cells), as determined using western blotting. C) Micronucleus rate in HCT116 cells transiently overexpressing *YY2* at indicated time-points after doxycycline-withdrawal, as stained using DAPI. Representative images of micronuclei (indicated by arrowheads; scale bars: 20 μm) and quantification results (ratio of micronuclei number to total cell number; each dot represents micronucleus rate/slide with > 100 cells/slides; four technical replicates from three independent experiments) are shown. D) Colony-formation potential of TetOn-*YY2* Trans cells at 10 days. Representative images (left) and quantification results (right; each dot represents the mean value of three technical replicates) are shown. E) Micronucleus rate of reversine-treated TetOn-*YY2* Trans cells 48 h after sorting. Representative images of micronuclei (indicated by arrowheads; scale bars: 20 μm) and micronucleus rate (ratio of micronuclei number to total cell number; each dot represents micronucleus rate/slide with > 100 cells/slides; four technical replicates from three independent experiments) are shown. F–G) Viability (F) and cell death rate (G) of reversine-treated TetOn-*YY2* Trans cells at indicated times and 5 days after oxaliplatin treatment, respectively. Cells infected with lentivirus generated using pTRIPZ-control (Con) were used as control. β-actin was used for western blotting loading control. Quantification data are shown as mean ± SD. All data were

obtained from three independent experiments. *P* values were calculated by one-way ANOVA. TetOn-*YY2* Trans and Conti: cells infected with TetOn-*YY2* lentivirus and treated with doxycycline only before sorting or continuously, respectively; Dox: doxycycline (final concentration: 2 μg mL^{-1}); Rev: Reversine (final concentration: 0.2 μM); $^{**}P < 0.01$; NS: not significant.

Supplementary Figure 11

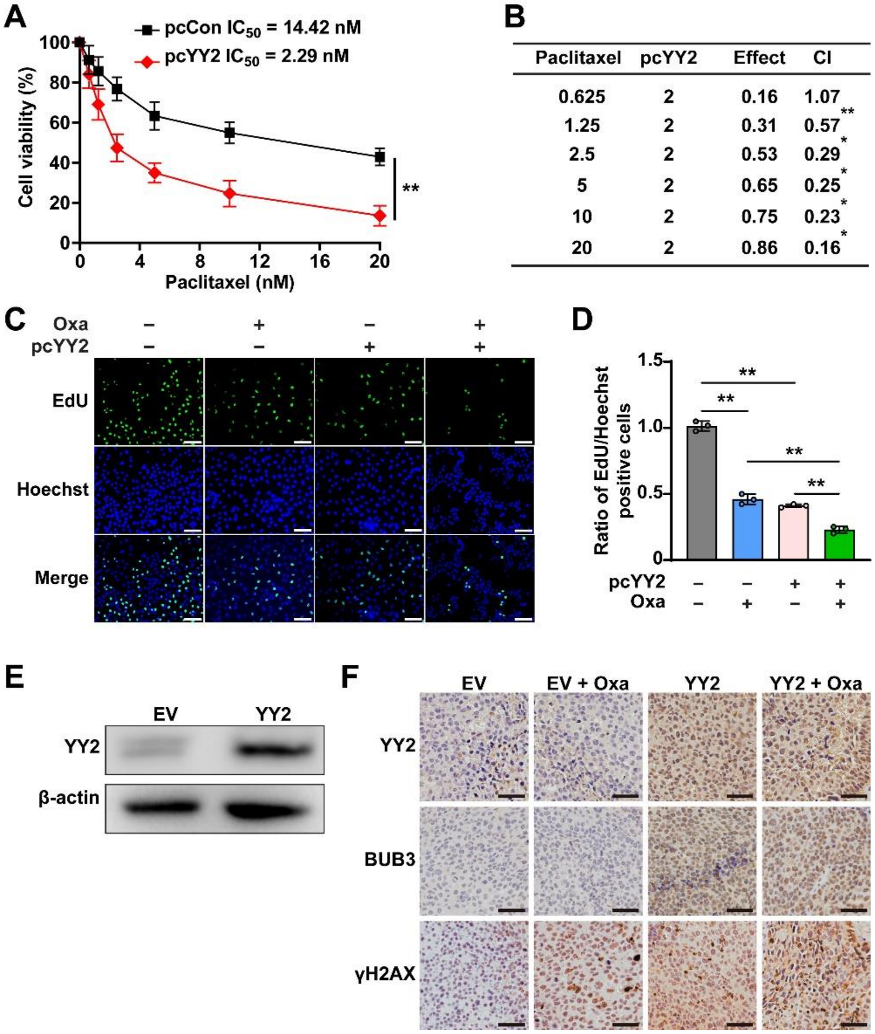

Paclitaxel	pcYY2	Effect	CI
0.625	2	0.16	1.07
1.25	2	0.31	0.57**
2.5	2	0.53	0.29*
5	2	0.65	0.25*
10	2	0.75	0.23*
20	2	0.86	0.16*

Figure S11. YY2 enhances DNA-damage inducing agent tumor suppressive activity. A) Viability of *YY2*-overexpressed HCT116 cells treated with indicated concentrations of paclitaxel for 48 h. IC_{50} was calculated using CompuSyn. B) Combination index (CI) between paclitaxel and *YY2* overexpression, as calculated using CompuSyn. C–D) Proliferation potential of oxaliplatin-treated *YY2*-overexpressed HCT116 cells as determined using EdU incorporation assay. Representative images (KC, scale bars: 200 μm) and ratio of proliferative cells (D; each dot represents the mean value of three technical replicates) are shown. E) YY2 protein expression level in HCT116 cells stably overexpressing *YY2* established using Lenti-YY2 virus, as determined using western blotting. F) IHC staining images against YY2, BUB3, and γH2AX in tissue sections of xenografted tumors (scale bars: 50 μm). Cells transfected with pcCon or infected with lentivirus generated using empty lentivirus (EV) were used as control. β-actin was used as western blotting loading control. Quantification data are shown as mean ± SD. All data were obtained from three independent experiments. *P* values were calculated by one-way ANOVA. Oxa: oxaliplatin; $^{**}P < 0.01$.

Supplementary Figure S12

A

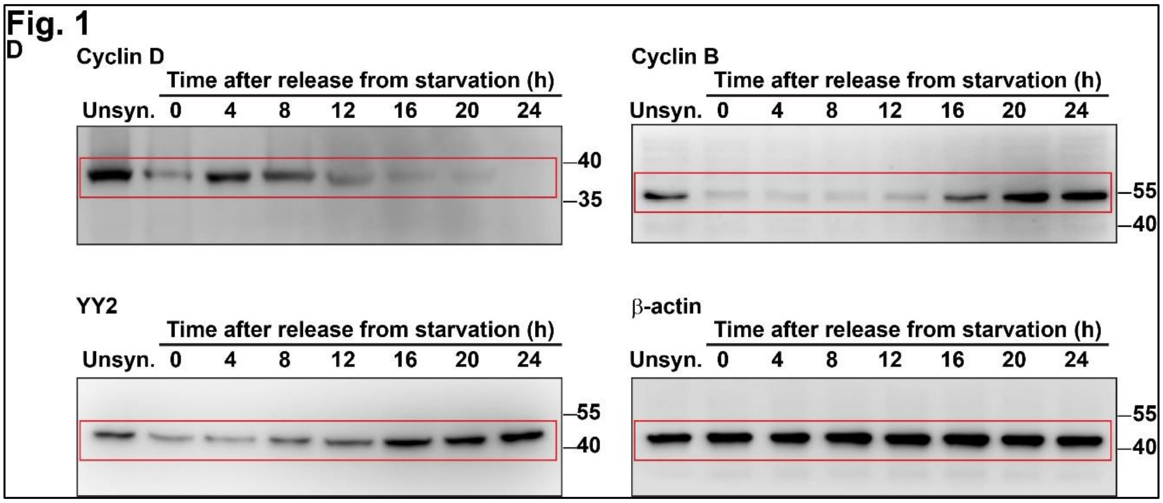

Figure S12. Uncropped western blots with the indicated areas of selection in Figs 1, 3, 7, and Supplementary Figs S1, S2, S3, S4, S5, S9, S10, and S11. (continued)

B

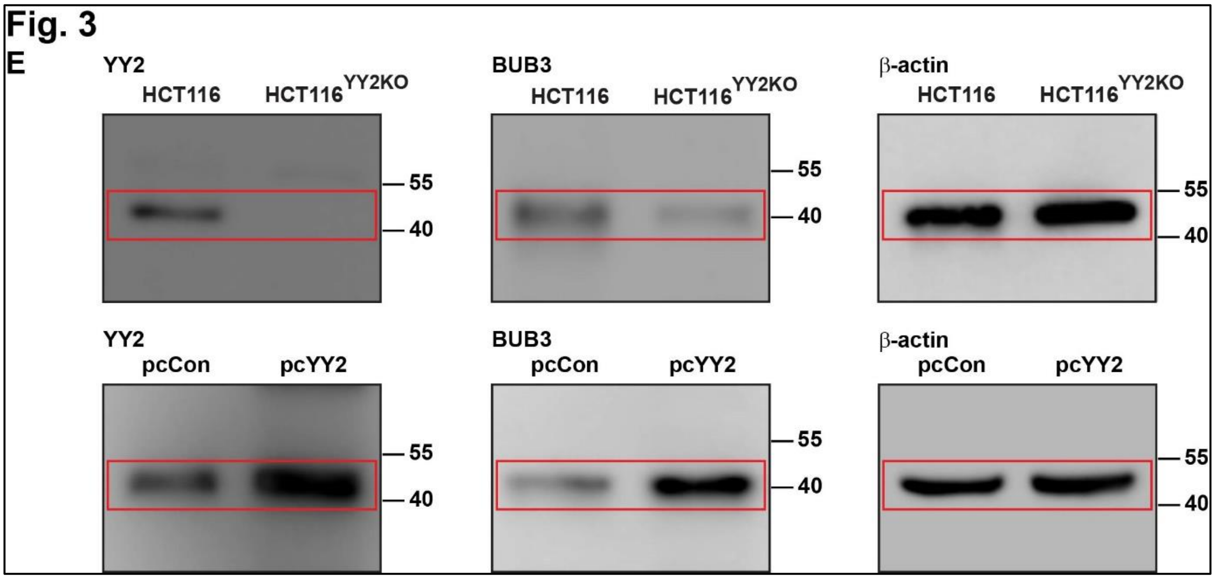

Figure S12. Uncropped western blots with the indicated areas of selection in Figs 1, 3, 7, and Supplementary Figs S1, S2, S3, S4, S5, S9, S10, and S11. (continued)

C

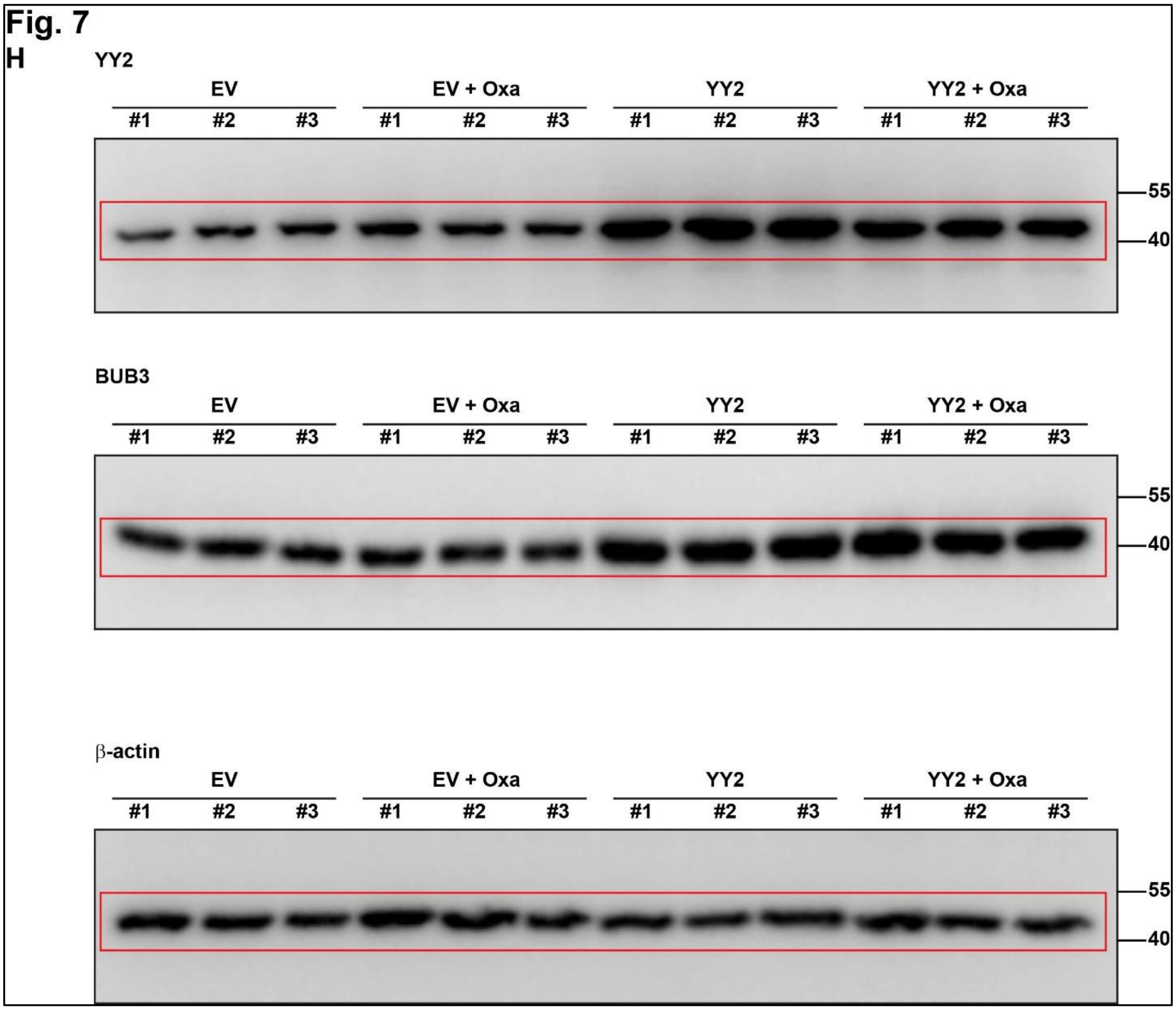

Figure S12. Uncropped western blots with the indicated areas of selection in Figs 1, 3, 7, and Supplementary Figs S1, S2, S3, S4, S5, S9, S10, and S11. (continued)

D

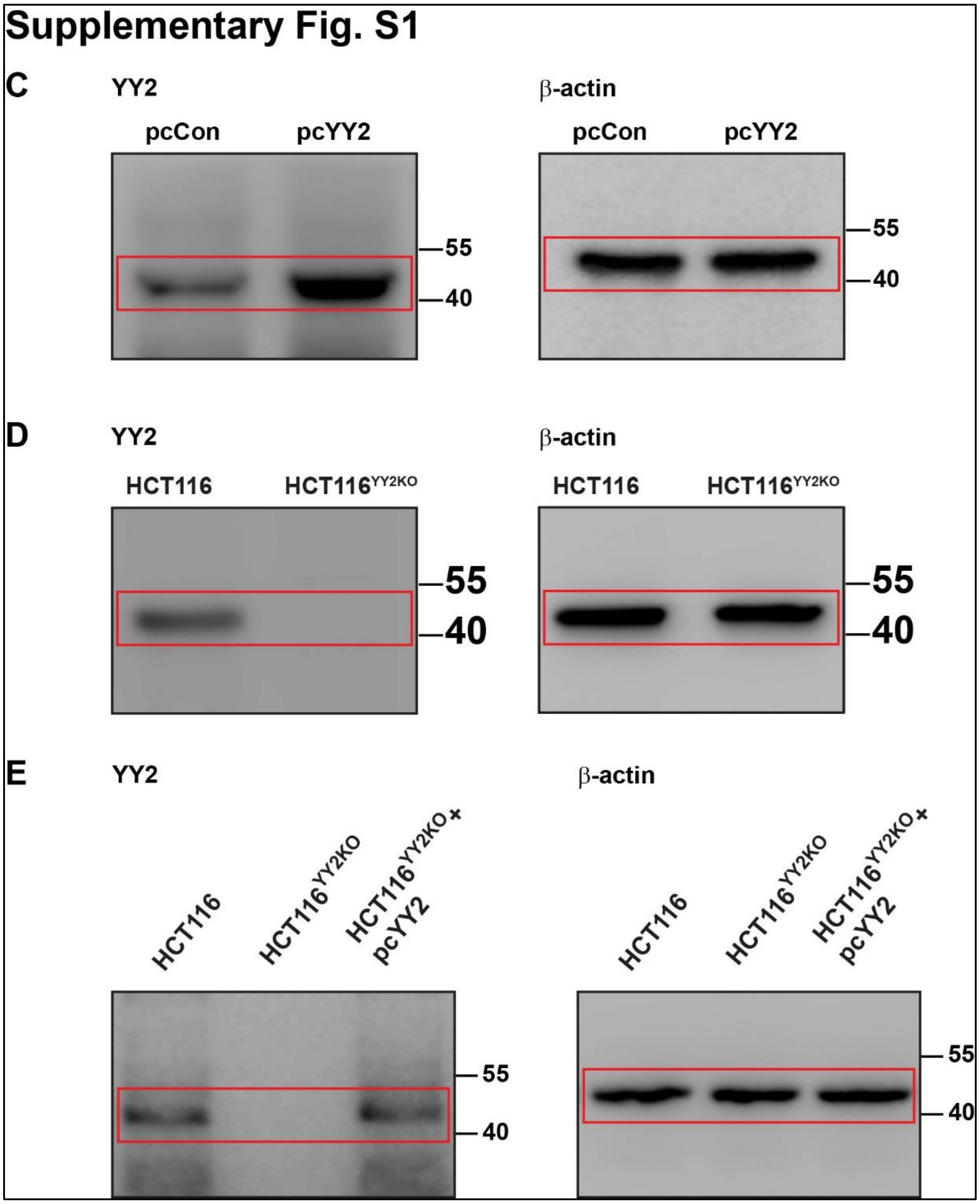

Figure S12. Uncropped western blots with the indicated areas of selection in Figs 1, 3, 7, and Supplementary Figs S1, S2, S3, S4, S5, S9, S10, and S11. (continued)

E

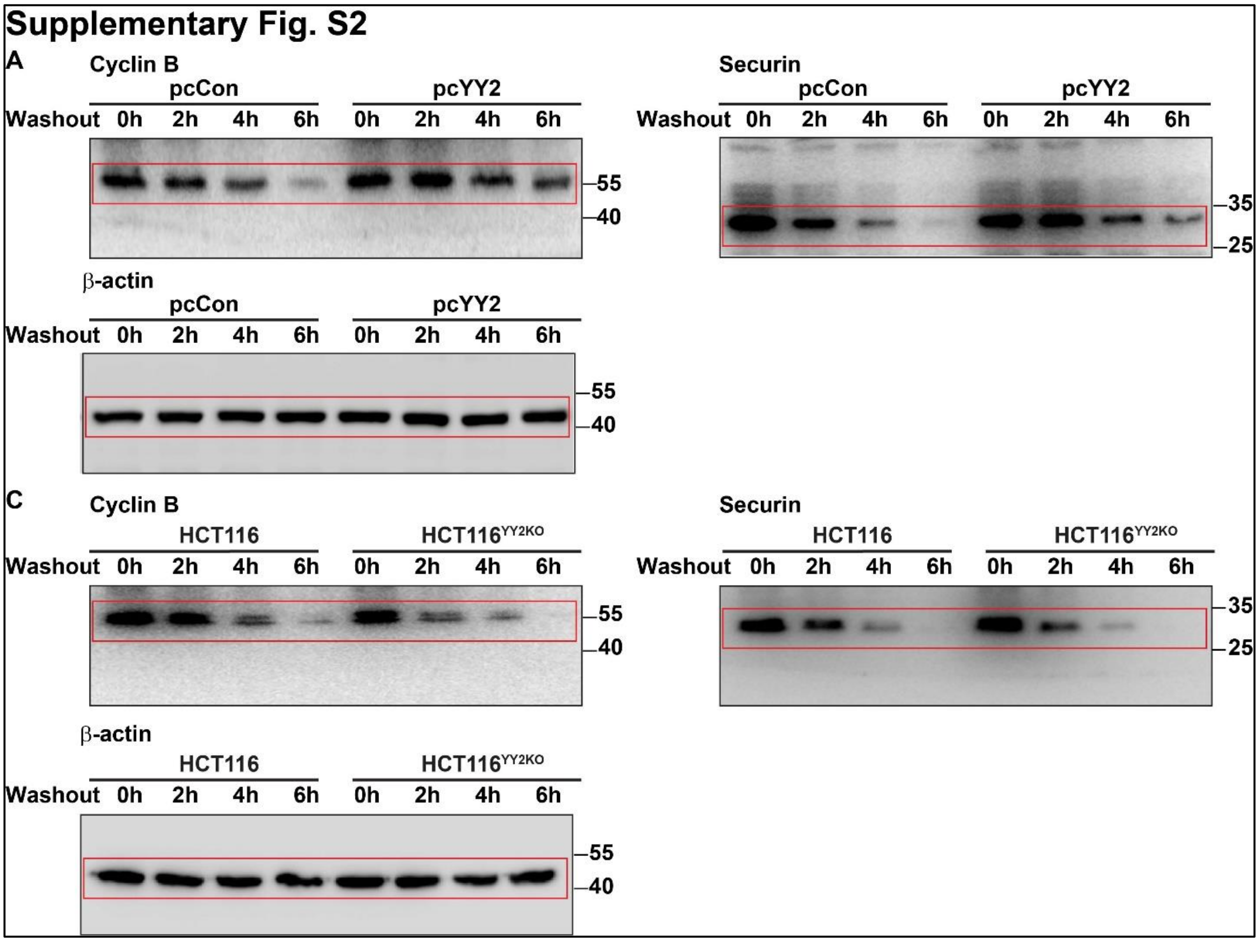

Figure S12. Uncropped western blots with the indicated areas of selection in Figs 1, 3, 7, and Supplementary Figs S1, S2, S3, S4, S5, S9, S10, and S11. (continued)

F

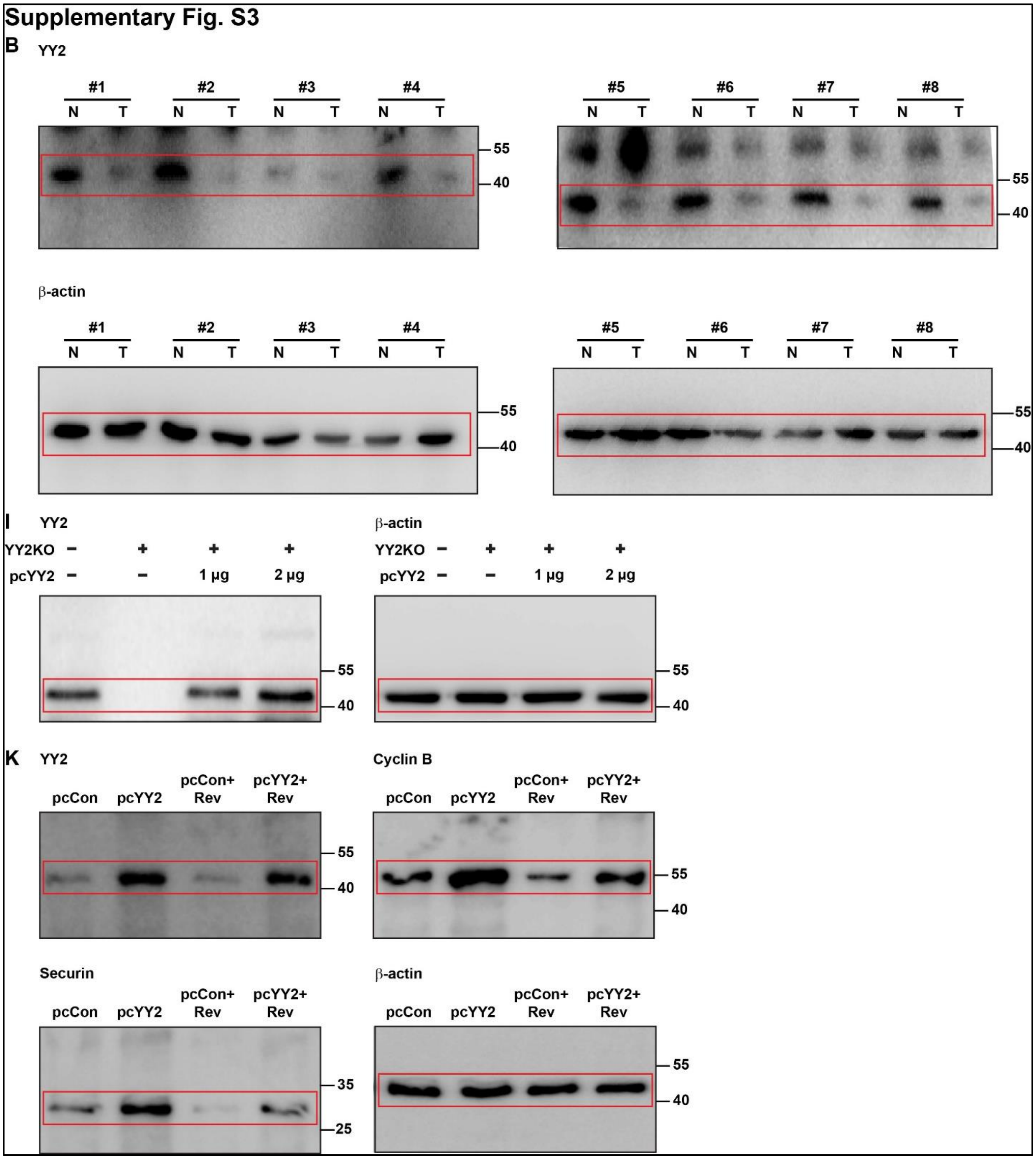

Figure S12. Uncropped western blots with the indicated areas of selection in Figs 1, 3, 7, and Supplementary Figs S1, S2, S3, S4, S5, S9, S10, and S11. (continued)

G

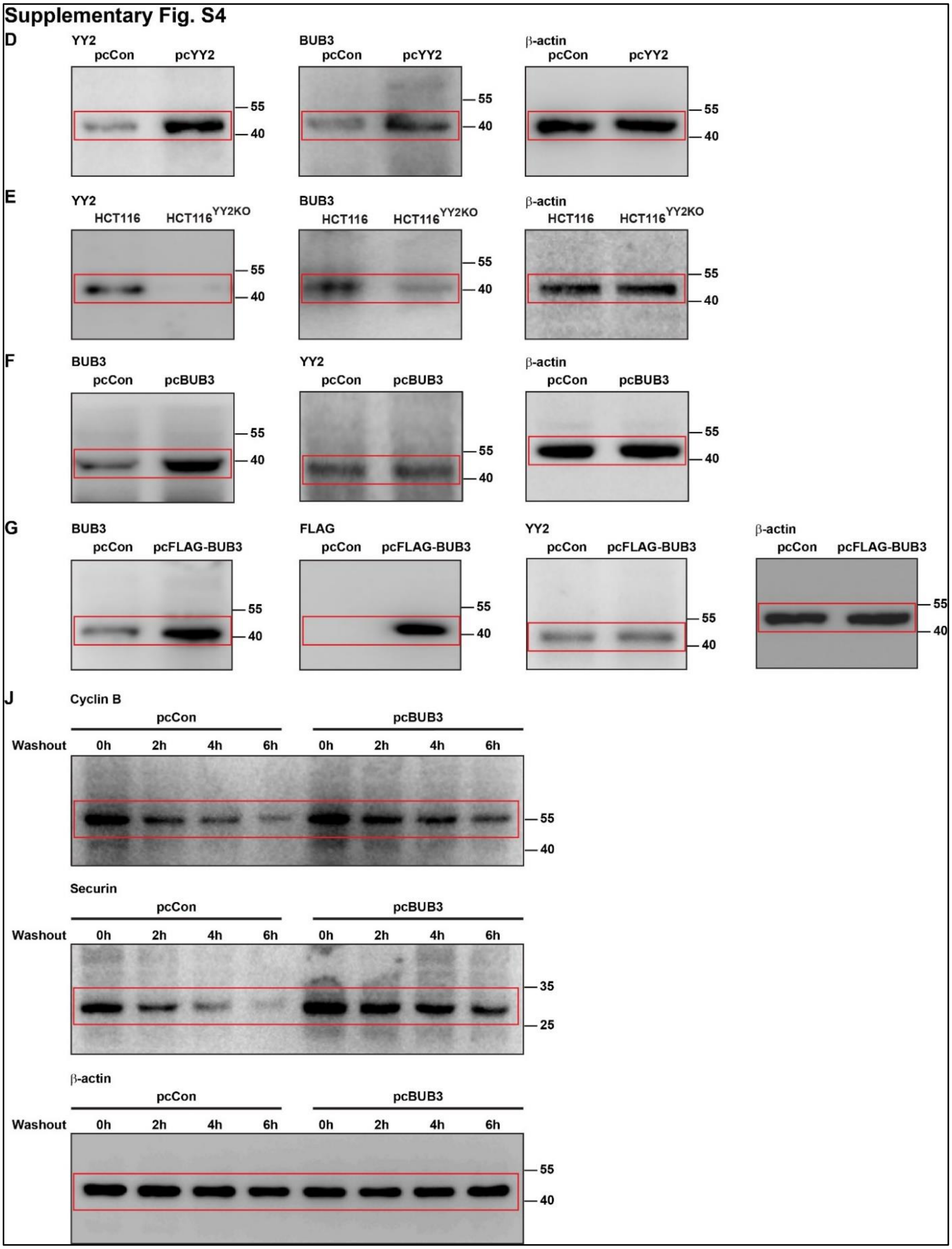

Figure S12. Uncropped western blots with the indicated areas of selection in Figs 1, 3, 7, and Supplementary Figs S1, S2, S3, S4, S5, S9, S10, and S11. (continued)

H

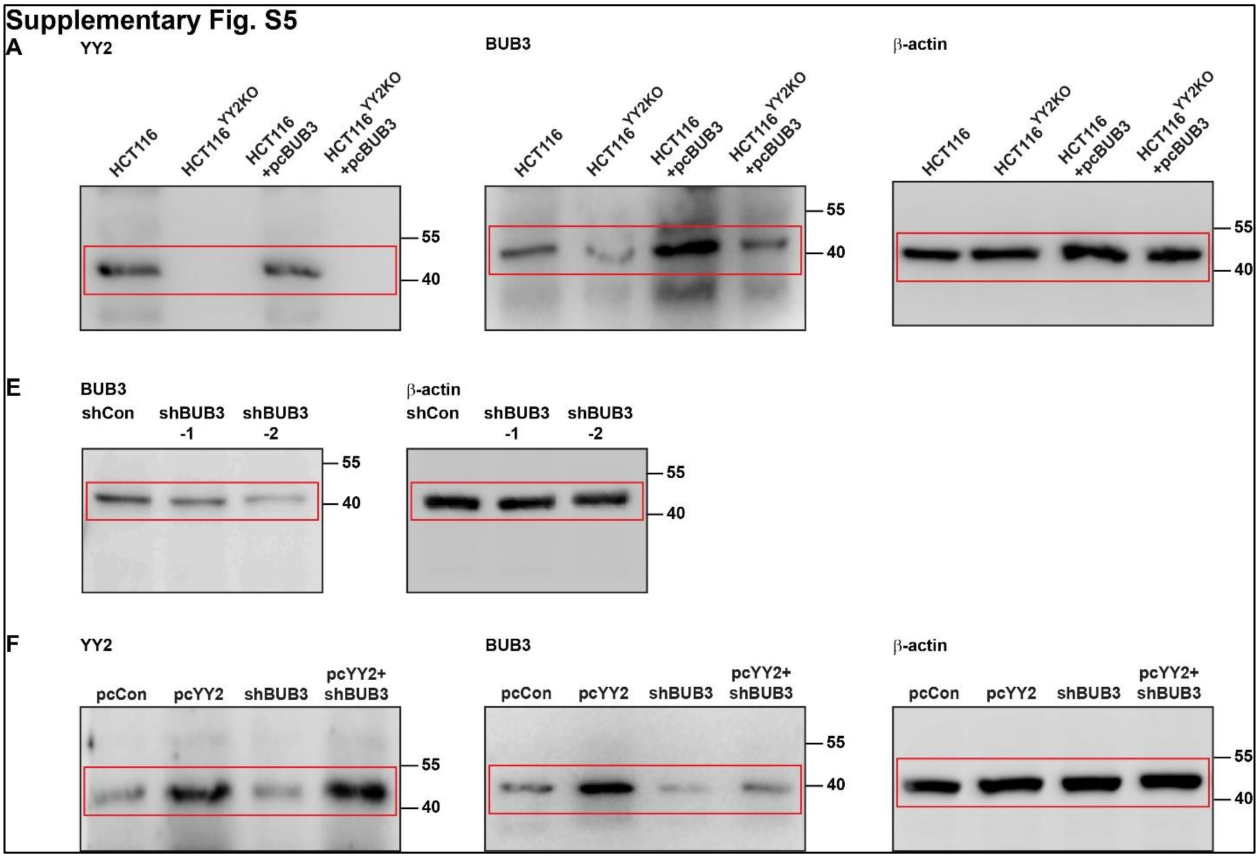

Figure S12. Uncropped western blots with the indicated areas of selection in Figs 1, 3, 7, and Supplementary Figs S1, S2, S3, S4, S5, S9, S10, and S11. (continued)

I

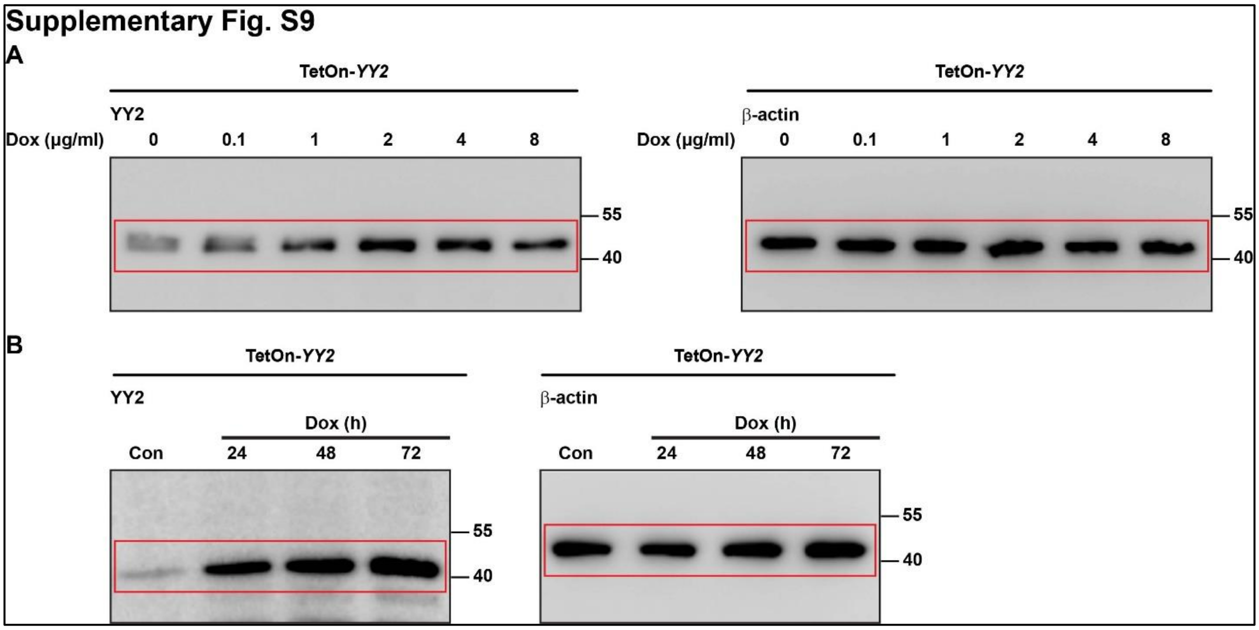

Figure S12. Uncropped western blots with the indicated areas of selection in Figs 1, 3, 7, and Supplementary Figs S1, S2, S3, S4, S5, S9, S10, and S11. (continued)

J

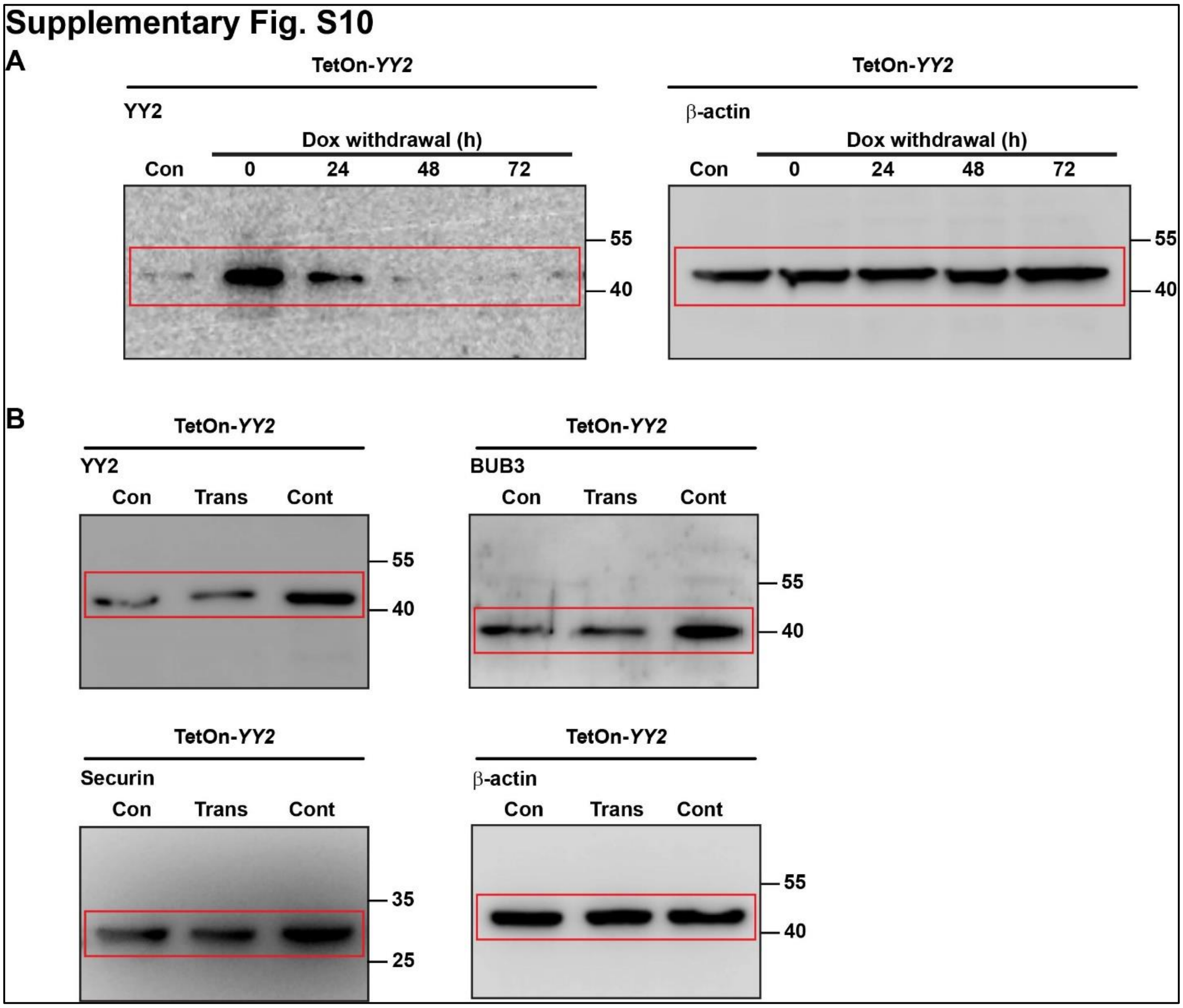

Figure S12. Uncropped western blots with the indicated areas of selection in Figs 1, 3, 7, and Supplementary Figs S1, S2, S3, S4, S5, S9, S10, and S11. (continued)

K

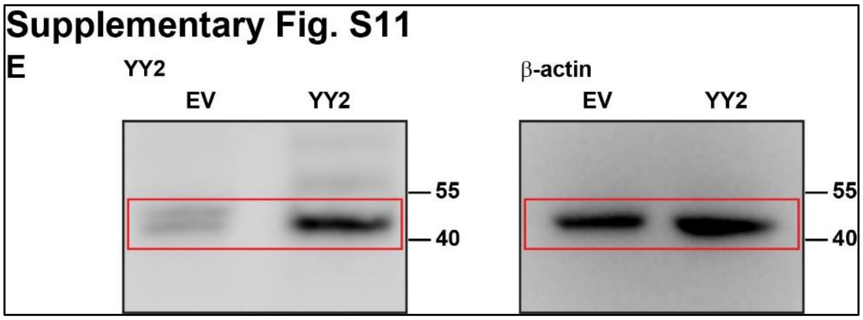

Figure S12. Uncropped western blots with the indicated areas of selection in Figs 1, 3, 7, and Supplementary Figs S1, S2, S3, S4, S5, S9, S10, and S11.

Table S1. Primer pairs used for qRT-PCR.

Gene	Refseq No.	Forward primer sequence (5'-3')	Reverse primer sequence (5'-3')
YY2	NM_206923.4	GCAGTGGGTGAAGGCCAGGCTG	CGGTGTGGACCAGCTGGTGTCG
BUB3	NM_004725.4	AATGCTGGGACCTTCTCTCA	TCCGTAAGTCCCACACCAAC
TEX14	NM_031272.5	AGTCCCCTGTCCTGTTCAACT	GCCGCAACAAAAAGTGCTGT
ZFP69	NM_001320178.2	CAAGGCCGATGTGAAGTGGA	CTCCACGGCCTTCTTTGGAT
ZFP69B	NM_001369565.1	TGACCCTTGGATCCCTGACA	TGGAAAGCTGACATCCCACT
ZNF93	NM_031218.4	ACTCTGTAACCATCCCAAACTCC	CCTCCAGGTCTCCGAAATGG
ZNF215	NM_001354853.2	CTCCTAGGGGGTTCCTTCCA	GGAGCTGGCGTTGGTAATCA
ZNF280C	NM_017666.5	ATTGCCTTTAATTGGCTCTCCTC	AGCTCTTAGGTAATCTGCTCCAG
ZNF730	NM_001277403.2	CCGCCGAAGCTCCAATTTTC	GGATCTCCCAATACCCGCAG
ZNF878	NM_001080404.3	GCTCGCGCTAGTTAAGGTCT	GGCCACCGAATCCATTTCCT
ZNF888	NM_001310127.2	CTTGTAGATTCGCCCGGACC	CACATACCCAGAGTCGCCG
FAM111B	NM_001142703.2	GCCCTTGAAATGCAGAATCCA	GCTGTAAACACACTACGGTCTAA
EGR1	NM_001964.3	TGATGTCCCCGCTGCAG	GTCCATGGTGGGCGAGTG
IFFO1	NM_001193457.2	CTGAACCTCCGGTTCCGCTGCTTC	GATGGGGCTGACGAAGCCGGTCTG
NR5A2	NM_001276464.2	AAGCGTTGTCCTTACTGTCG	CCCTGTCTCTCTTGTACATTGG
SHH	NM_000193.4	GAAAGCAGAGAACTCGGTGG	GGAAAGTGAGGAAGTCGCTG
SH3RF1	NM_020870	GGCGCGAGAGCAAAGT	ACCTACGATCCCCAGCAAAC
AMIGO2	NM_001370299.1	GCACGAAAGGAACCATTGAT	CCTTCATGGAAACCCATTTG
AKR1B1	NM_001628.4	TTTTCCCATTGGATGAGTCGG	CCTGGAGATGGTTGAAGTTGG
CHST11	NM_001173982.2	AAACGCCAGCGGAAGAA	GGGATGGCAGAGTGAGTAGA
β-Actin	NM_001101.3	CGAGCGCGGCTACAGCTT	TCCTTAATGTCACGCACGATTT

Table S2. Antibodies used for western blotting, immunohistochemistry, and ChIP assay.

**Antibody**	**Product No.**	**RRID**	**Maker**	**Experiment**	**Dilution**
anti-YY2	sc-374455	AB_10988247	Santa Cruz Biotechnology	Western blotting<br>ChIP<br>IHC	1/1,000<br>30 μg mL^{-1} cell lysate<br>1/100
anti-cyclin D1	sc-8396	AB_627344	Santa Cruz Biotechnology	Western blotting	1/1,000
anti-cyclin B1	sc-245	AB_627338	Santa Cruz Biotechnology	Western blotting	1/1,000
anti-BUB3	27073-1-AP	AB_2880743	Proteintech	Western blotting<br>IHC	1/5,000<br>1/100
anti-FLAG	F1804	AB_262044	Sigma Aldrich	Western blotting	1/1,000
anti-securin	sc-56207	AB_785382	Santa Cruz Biotechnology	Western blotting	1/500
anti-γH2AX	GB111841		Servicebio	IHC	1/100
anti-β-actin	66009-1-Ig	AB_2687938	Proteintech	Western blotting	1/50,000
Goat Anti-Rabbit IgG	ZB2301	AB_2747412	ZSGB-BIO	Western blotting	1/10,000
Goat Anti-Mouse IgG	ZB2305	AB_2747415	ZSGB-BIO	Western blotting	1/10,000

Video Legend

Video S1. Time-lapse video of control HCT116 cells related to Fig. 1. The display rate is one frame every 200 millisecond. Still images of this video are shown in Fig. 1E.

Video S2. Time-lapse video of *YY2*-overexpressed HCT116 cells related to Fig. 1. The display rate is one frame every 200 millisecond. Still images of this video are shown in Fig. 1E.

Video S3. Time-lapse video of wild-type HCT116 cells related to Fig. 1. The display rate is one frame every 200 millisecond. Still images of this video are shown in Fig. 1F.

Video S4. Time-lapse video of HCT116^{YY2KO} cells related to Fig. 1. The display rate is one frame every 200 millisecond. Still images of this video are shown in Fig. 1F.

Video S5. Time-lapse video of *YY2*-overexpressed, reversine-treated HCT116 cells related to Fig. 2. The display rate is one frame every 200 millisecond. Still images of this video are shown in Fig. 2C.

Video S6. Time-lapse video of *BUB3*-overexpressed HCT116 cells related to Fig S4. The display rate is one frame every 200 millisecond. Still images of this video are shown in Fig S4H.

Video S7. Time-lapse video of *BUB3*-overexpressing HCT116^{YY2KO} cells related to Fig S5. The display rate is one frame every 200 millisecond. Still images of this video are shown in Fig S5B.

Video S8. Time-lapse video of *BUB3* knocked-down HCT116 cells related to Fig S5. The display rate is one frame every 200 millisecond. Still images of this video are shown in Fig S5G.

Video S9. Time-lapse video of *BUB3* knocked-down, *YY2*-overexpressed HCT116 cells related to Fig S5. The display rate is one frame every 200 millisecond. Still images of this video are shown in Fig S5G.

Video S10. Time-lapse video of reversine-treated HCT116^{YY2KO} cells related to Fig S9. The display rate is one frame every 200 millisecond. Still images of this video are shown in Fig S9C.
